# Geochemical Evidence of First Forestation in the Southernmost Euramerica from Upper Devonian (Famennian) Black Shales

**DOI:** 10.1038/s41598-019-43993-y

**Published:** 2019-05-20

**Authors:** Man Lu, YueHan Lu, Takehito Ikejiri, Nicholas Hogancamp, Yongge Sun, Qihang Wu, Richard Carroll, Ibrahim Çemen, Jack Pashin

**Affiliations:** 1 Department of Geological Sciences, Alabama Water Institute, University of Alabama, Tuscaloosa, AL 35485 USA; 2 Alabama Museum of Natural History, University of Alabama, Tuscaloosa, AL 35485 USA; 3 Hess Corporation, 1501 McKinney Street, Houston, TX 77010 USA; 4 Environmental and Biogeochemical Institute (ebig), School of Earth Science, Zhejiang University, Hangzhou, 310027 China; 5 Key Laboratory of Water Quality and Conservation of the Pearl River Delta, Ministry of Education, Institute of Environmental Research at Greater Bay, Guangzhou University, Guangzhou, 510006 China; 6 Energy Investigation Program, Geological Survey of Alabama, Tuscaloosa, AL 35401 USA; 7 Boone Pickens School of Geology, Oklahoma State University, Stillwater, OK 74078 USA; 8 Academy for Advanced Interdisciplinary Studies, Southern University of Science and Technology, Shenzhen, Guangdong 518055 China

**Keywords:** Biogeochemistry, Palaeoclimate

## Abstract

The global dispersal of forests and soils has been proposed as a cause for the Late Devonian mass extinctions of marine organisms, but detailed spatiotemporal records of forests and soils at that time remain lacking. We present data from microscopic and geochemical analyses of the Upper Devonian Chattanooga Shale (Famennian Stage). Plant residues (microfossils, vitrinite and inertinite) and biomarkers derived from terrestrial plants and wildfire occur throughout the stratigraphic section, suggesting widespread forest in the southern Appalachian Basin, a region with no macro plant fossil record during the Famennian. Inorganic geochemical results, as shown by increasing values of SiO_2_/Al_2_O_3_, Ti/Al, Zr/Al, and the Chemical Index of Alteration (CIA) upon time sequence, suggest enhanced continental weathering that may be attributed to the invasion of barren lands by rooted land plants. Our geochemical data collectively provide the oldest evidence of the influences of land plants from the southernmost Appalachian Basin. Our synthesis of vascular plant fossil record shows a more rapid process of afforestation and pedogenesis across south-central Euramerica during the Frasnian and Famennian than previously documented. Together, these results lead us to propose a new hypothesis that global floral dispersal had progressed southward along the Acadian landmass rapidly during the Late Devonian.

## Introduction

The Late Devonian is known for the rapid and global radiation of early forests and soils such as spodosols and alfisols^1–3^. The development of land plants and soils is hypothesized to have been either a trigger or consequence of a series of global changes in the lithosphere (e.g., increased weathering and erosion), hydrosphere (e.g., anoxic oceans, global transgression and regression), atmosphere (e.g., global changes in O_2_ and CO_2_), and biosphere (i.e. mass extinctions of marine life) during the Middle to Late Devonian^4–10^. One compelling hypothesis is that forest radiation mobilized a tremendous amount of soils and associated nutrients (N and P) to coastal oceans for the first time in Earth’s history and led to dysoxic/anoxic oceans globally^4,11–13^. Testing this hypothesis, however, requires data on stratigraphic occurrences of soils and plants in specific paleogeographic areas.

**Figure 1:**
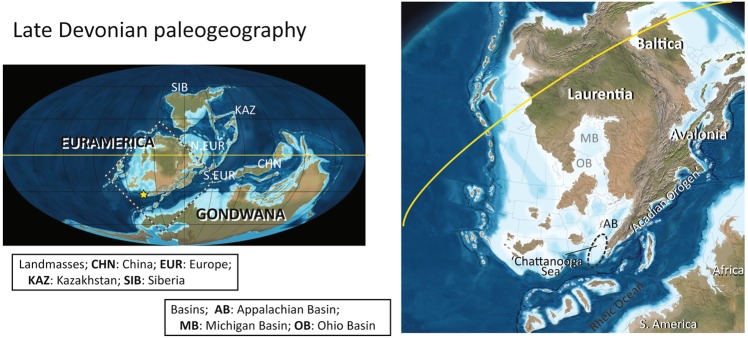
Late Devonian paleogeography. Left: the global scale; right: south-central Euramerica. The yellow star indicates the study site (the present location of Alabama), which represents the southern part of the Chattanooga Sea (dashed elliptical area). Maps are adapted from original map (360 Ma) from Global Paleogeography and Tectonics in Deep Time Series by Ron Blakey [© 2016 Colorado Plateau Geosystems Inc.].

Current knowledge on the paleogeographic distribution of Devonian forests is largely based on macrofossils, such as tree trunks, stems, leaves, and roots, as well as some microfossils such as spores^14–17^. The oldest tree stems and stumps in the Euramerica are reported from the uppermost Givetian (Middle Devonian) strata of New York (i.e., Gilboa Park and Cairo) representing the central Euramerican landmass^18^ (Fig. 1). To date, the record of Devonian trees and shrubs assigned to pteridophytes, which were likely the primary component for the first forest, is very limited from the southern Appalachian Basin along the southern Acadian landmass. Only a few uncertain remains have been reported as small wood fragments of possible *Callixylon*^19^ and *Foerstia*^20^ from Tennessee. In contrast, some tree or shrub fossils are known from the northern Appalachian Basin (e.g., New York, Pennsylvanian, West Virginia) and the Baltica and Avalonia landmasses (e.g., United Kingdom, Belgium)^21–27^. This gap in the paleogeographic occurrence between the northern and southern parts of the Appalachian Basin implies that forests originated from the central Euramerica in the late Middle Devonian and dispersed southward during the Late Devonian. Better understanding of the spatiotemporal occurrence of land plants in the southern Appalachian Basin will provide better understanding of the dispersal pattern of early land forest.

To date, fluvial sedimentary sequences are the most frequently reported units containing tree and shrub fossils (lycopsids, cladoxylopsids, progymnosperms, and possibly stem spermatophytes) and soils in the Appalachian Basin^1,18,28,29^. Those sandstone, siltstone, and limestone deposits are geographically distributed along the eastern side of the basin (close to the modern-day Appalachian Mountains)^30^. By comparison, very few tree fossils have been reported from the extensive Upper Devonian black shale deposits further offshore from the western margin of the Appalachian Basin, including the Ohio Shale, New Albany Shale, Cleveland Shale, Huron-Dunkirk shales, Millsboro Shale, and Chattanooga Shale. Typically, the preservation of land plant fossils in offshore marine deposits is not as common as that in nearshore environments (e.g., fluvial, deltaic), because plant remains can be easily broken into pieces and decomposed through taphonomic process. However, if information on the distribution of early forests could be obtained from the abundant and regionally extensive Upper Devonian marine black shales, it would significantly increase the amount of data on the occurrence of land plants available to geologists from the Paleozoic rock record. Such efforts would allow for the reconstruction of a far more spatially and stratigraphically detailed record of afforestation than is possible using the rarely preserved fluvial deposits alone.

In the present study, we present identifiable signatures of forests and soils preserved in unfossiliferous black shales in the southernmost Appalachian Basin. We further demonstrate the potential of using these signatures to generate new understanding of the dispersal patterns of Famennian forest (land plant) and pedogenesis along the southern Acadian Orogen. We analyzed a complete section of the Chattanooga Shale in northeastern Alabama (Fig. 1). The Chattanooga Shale and other Upper Devonian black shale units in the Appalachian Basin are interpreted to have accumulated in a basin-like depositional environment further offshore than equivalent sandstone or siltstone dominated formations (i.e., alluvial plain or basin margin-like environment)^30,31^. Plant macrofossils such as stems, leaves, stumps, and roots are nearly absent in the Chattanooga Shale, apart from a few brief notes from central Tennessee^19^. Using microscopic investigation and a comprehensive set of geochemical analyses (e.g., inertinite and vitrinite, mineral composition, trace metals, stable carbon isotope ratio, and biomarker assemblages), we investigate whether early forests had left detectable signals in the Chattanooga Shale of Alabama. The multiple geochemical-tracer approach we use here can overcome the preservation limit of macrofossils, and thus extend our knowledge of the spatiotemporal pattern of the early forest radiation in the southernmost Euramerican continent. Our data can set the foundation for new hypotheses regarding how afforestation progressed during the Late Devonian.

## Results

### Geological background

**Figure 2:**
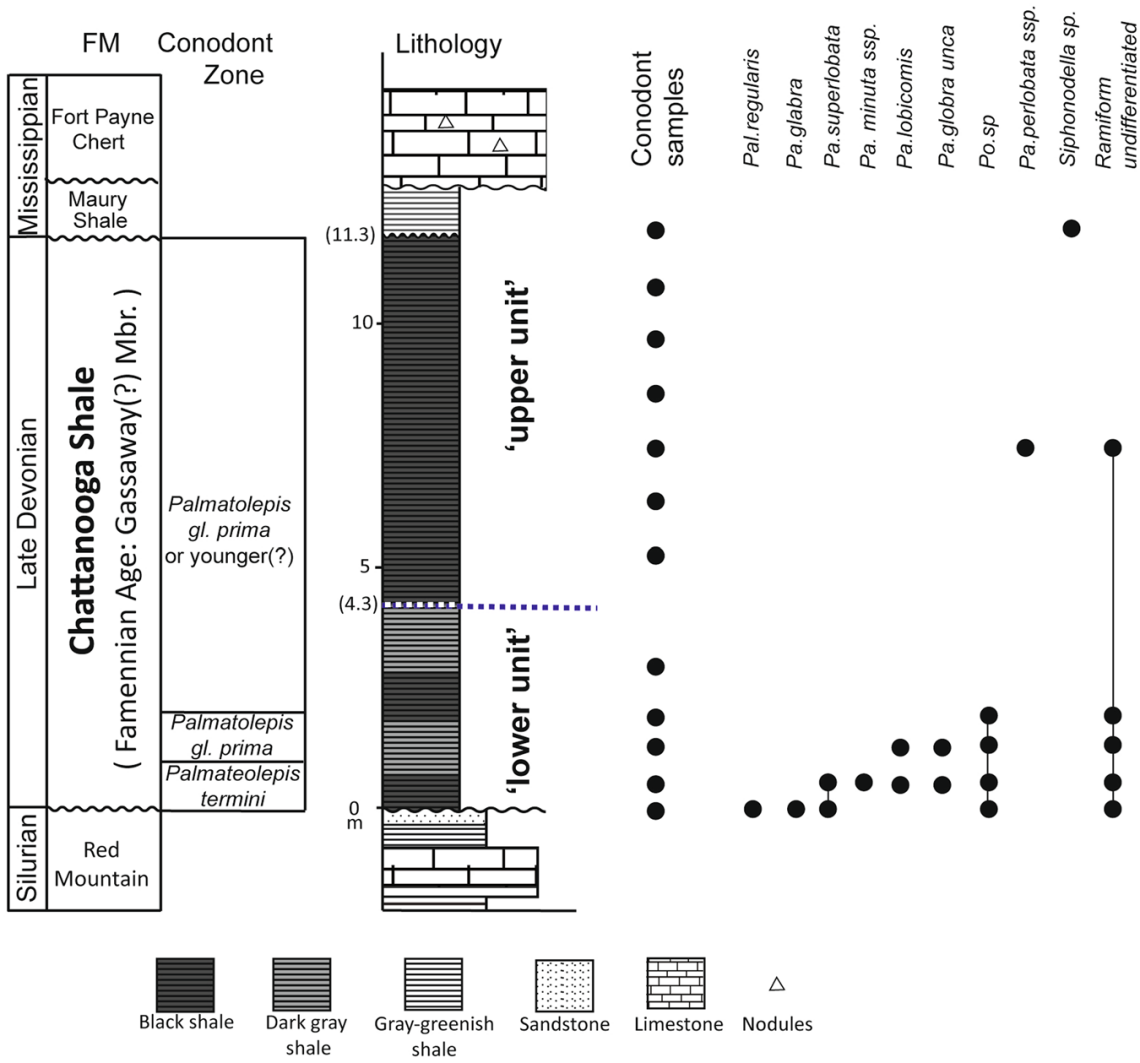
Stratigraphic column and conodont occurrence of the Upper Devonian Chattanooga Shale in northeastern Alabama. The site is located in Fort Payne, northeastern Alabama. The conodont biozone is based on refs^36,40^.

The outcrop of the Chattanooga Shale for this study is located in DeKalb County, northeastern Alabama (Fig. 1) (Material and Methods). The Chattanooga Shale is exposed with disconformable boundaries above the Upper Silurian Red Mountain Sandstone and below the Lower Mississippian Maury Shale. The Chattanooga Shale in this outcrop is 11.3 m thick and is subdivided here into a lower and upper unit based on lithological characteristics (Fig. 2). The lower unit is 4.3 m thick and characterized by thinly laminated, pyritic, fissile rocks composed of layers of interbedded gray and black shale. The upper unit is 7.0 m thick and is composed of dark gray to black, silty, blocky shales. Similar stratigraphic features have been reported from other Chattanooga Shale sections, entirely or in part, in northeastern Alabama^32,33^.

No macrofossils (visible to the naked eye) have been reported from the Chattanooga Shale in Alabama, aside from a single bivalve shell from a different nearby section (personal observation in 2015). Detailed field work indicates that the studied outcrop is largely unfossiliferous (i.e., no macrofossils were observed with the naked eyes), confirming previous studies. In contrast, macrofossils of marine invertebrates (e.g., brachiopods, crinoids, corals) are relatively common in the Chattanooga Shale in Tennessee^20,31^. A few possible plant remains have also been reported from Tennessee, but, to date, nothing that has been identified to a specific taxon has been documented from Alabama or Tennessee^19^.

### Biostratigraphy

The phosphatic tooth-like remains of conodonts can be found in almost all marine deposits from the Lower Paleozoic to the Triassic^34^. For this reason, conodonts are the primary biostratigraphic fossils in most Paleozoic stages, including the Upper Devonian^35–39^. Conodonts were identified on bedding plane breaks throughout the studied outcrop to provide a biostratigraphic framework to constrain the timing of geochemical variation. The zonation names used in this section are from the updated Famennian zonation of Spalletta *et al*. (ref.^39^). For clarity and for easier comparison to previous studies (e.g., ref.^35^), the older zonation names are also included in parenthesis.

Most conodonts observed in the study outcrop of the Chattanooga Shale are external molds (Supplementary Fig. S6). Some phosphatic remains of conodont elements are present, but are often weathered, resulting in a white, partially dissolved element. Conodonts are most abundant in the lower unit, within the lowermost 1–2 m interval (Fig. 2). The absence of *Palmateolepis glabra unca* and *P*. *lobicornis* combined with the presence of *P*. *superlobata* in the 0–0.75 m interval indicates *Palmateolepis termini* Zone (Middle *crepida* Zone) in age. P_1_ elements of *P*. *lobicornis*, *P*. *minuta*, *P*. *superlobata*, and *P*. *glabra unca* were identified from the 1.25 m and 1.75 m levels, suggesting that this interval is *Palmatolepis glabra prima* Zone (Upper *crepida* Zone) in age^35,39^.

Conodonts were found through the study section. Although those remains tend to be scattered or poorly preserved in the upper unit (except well-preserved *Palmateolepis perlobata* at the 7.5 m-level), relatively abundant materials were recovered from the lower unit (esp., in the 0 m to 5 m interval from the base) (Fig. 2). Tasmanitid algal cysts are abundant from 5.5 to 7.5 m, and much more silty bedding planes were observed from 7.5 m to the top. Some skeletal fragments were observed under a Scanning Electron Microscope (SEM) that may be derived from brachiopods, bivalves, gastropods, and/or probable spicules of sponges. The abundant Famennian conodonts observed within the basal 2 m of the outcrop show that the studied section comprises only the Famennian Gassaway Member, and that the older Frasnian age Dowelltown Member is missing. Furthermore, the two lithological units identified in the study section resemble the lower and middle units of the Gassaway Member of the Chattanooga Shale in central Tennessee described previously^19,40^.

### Plant residue

**Figure 3:**
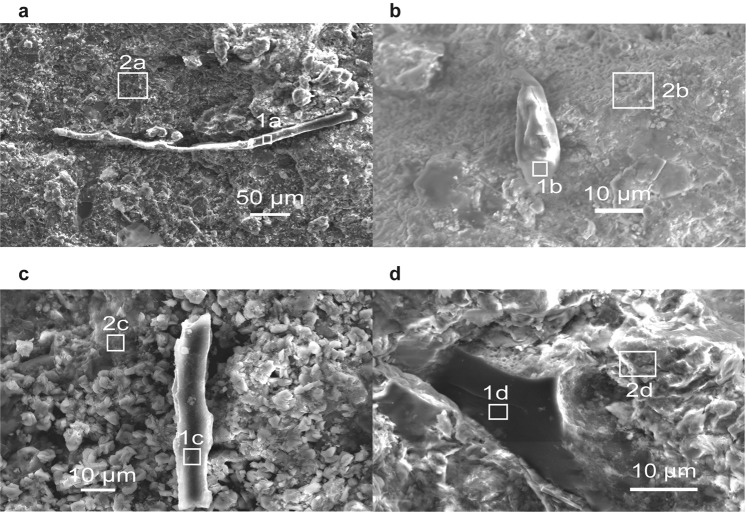
Scanning electron microscope (SEM) images of land plant remains from the Upper Devonian Chattanooga Shale in northeastern Alabama. (**a**) A tubular particle from the lower unit; (**b**) a deformed trilete spore from the upper unit; (**c**) a piece of wood fragment; and (**d**) a wood fragment with conducting tissues inside. The associated energy-dispersive X-ray spectroscopy (EDS) spectra are presented in Supplemental Material S7.

Plant residue were identified under microscopy. Those include fragments of leaves, branches, roots, and spores. Tubular or irregularly shaped, carbon-rich fragments are more common in the lower unit (Figs 3, Supplementary Fig. S7). Those tubular fragments display an overall curved shape with a smooth cortex. Spore-like particles and woody fragments were only observed in the upper unit. One well-preserved piece is elongated with a dimension of 30 μm in length and 10 μm in width and likely represents the remains of a spore. Spore-like particles have a rounded or elliptical shape with a bulged surface and are likely derived from trilete spore. Woody fragments are typically stick-like in shape, have a smooth surface, and have dimensions of 20–70 μm in length and about 10 μm in width. Some fragments with xylem- or phloem-like structures indicate functionally conducting wood tissues.

**Figure 4:**
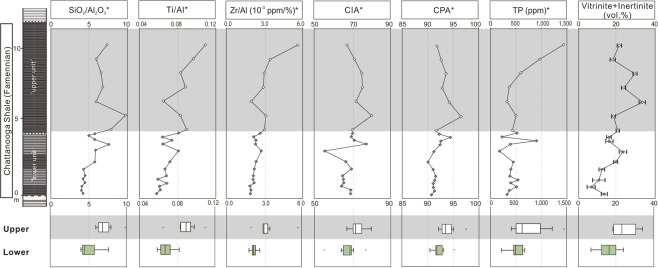
Variations in inorganic and palynological proxies across the Upper Devonian Chattanooga Shale in northeastern Alabama. Light grey color marks the upper unit of the section. Box plots show comparison of upper vs. lower unit, and asterisks indicate that significance is detected between the lower and upper units (α = 0.05). The higher values of SiO_2_/Al_2_O_3_ and vitrinite + inertinite in the upper unit reflect enhanced terrestrial plant inputs accompanied by high siliciclastic input. Correspondingly, the weathering indices (CIA and CPA) values are higher in the upper unit, reflecting enhanced weathering of land materials. The higher Ti/Aland Zr/Al values in the upper unit suggest increased contributions of heavy detrital sediment inputs, and the higher TP contents in the upper unit likely indicate a higher marine primary productivity.

Organic petrographic analyses show that more than 50% of macerals are amorphous organic matter (Supplementary Fig. S8) that is generally considered to be a degradation product of organic materials of mainly marine origin^41,42^. Figured components are mainly vitrinite and inertinite, which are commonly used as an indicator of plant residue in both fluvial and marine strata (e.g., refs^43–48^). In our samples, the majority of inertinite fragments are of high reflectance and sharply angular in shape (Fig. 4) that are suggested as typical features resulting from plant combustion^41,49^. The relative abundance of vitrinite + inertinite is hence used to represent terrestrial inputs. Our data show a significantly increasing trend from the lower unit (mean ± standard deviation = 15.6 ± 5.4%) to the upper unit (24.5 ± 6.4%) (Mann-Whitney U test: *P* = 0.026).

### Bulk and molecular characteristics of organic matter

**Figure 5:**
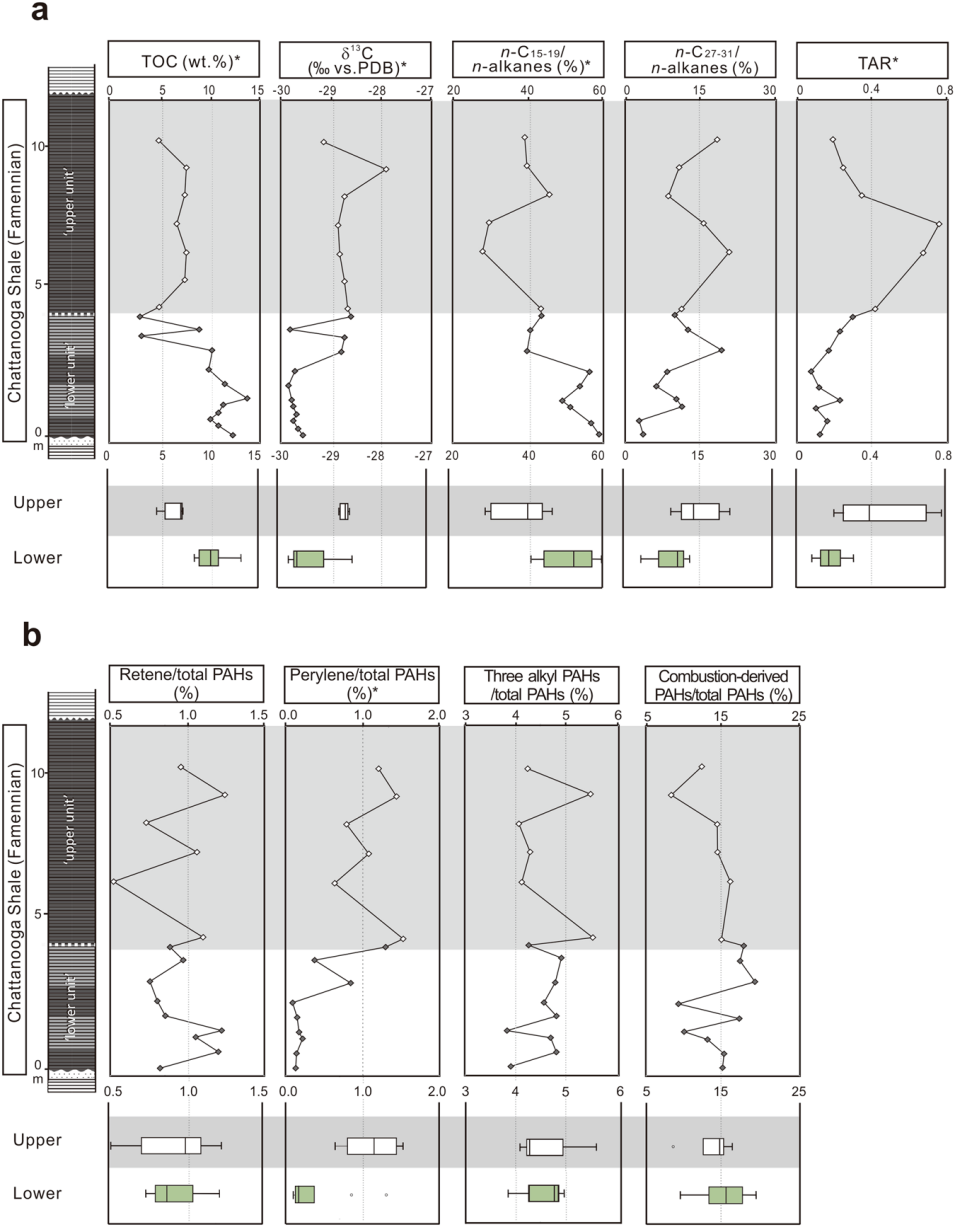
Variations in bulk and molecular organic proxies across the Upper Devonian Chattanooga Shale in northeastern Alabama. Light grey color marks the upper unit. Box plots show comparison of upper vs. lower unit, and asterisks indicate that significance is detected between the lower and upper units (α = 0.05). TOC contents are lower in the upper unit, reflecting the dilution by detrital, inorganic materials. Higher δ^13^C values in the upper unit may reflect increased phytoplankton growth. Normal alkane parameters all show a significantly higher contribution of higher land plant-derived organic matter in the upper unit, and PAHs from higher plants or plant combustion are present in all samples, indicating the contributions of organic matter from higher plants throughout the Famennian.

Total organic carbon (TOC) content of the rocks in the section range from 2.8% to 13.7% (Fig. 5). The TOC of black shales averages 9.6 ± 3.3% in the lower unit, which is significantly higher than that in the upper unit (6.6 ± 1.3%) (Mann-Whitney U test: *P* = 0.017). The δ^13^C values of TOC fluctuate between −29.9 and −27.9‰ with a significantly high value in the upper unit (−28.7 ± 0.4‰) than in the lower unit (−29.5 ± 0.5‰) (Mann-Whitney U test: *P* = 0.01).

**Figure 6:**
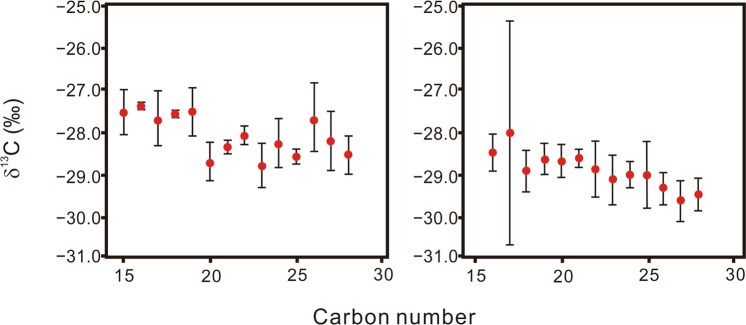
δ^13^C values of individual normal alkanes of two representative samples from the Upper Devonian Chattanooga Shale in northeastern Alabama. The left and right panels show samples from the upper and lower units, respectively. The δ^13^C values of short chain alkanes are more positive than those of long chain alkanes, supporting our interpretation that the two homologues represent different source organisms. Error bars represent standard deviation of replicate measurements.

The distribution of normal alkanes shows a carbon range from *n*-C_13_ to *n*-C_32_ with an enrichment in low molecular weight *n*-alkanes (*n*-C_15_ − *n*-C_19_: 45 ± 9.4% of total *n*-alkanes), which is typical for marine black shales^50–52^. The δ^13^C values of *n*-alkanes of selected samples demonstrate that short chain *n*-alkanes are on the average of 0.71‰ more enriched in ^13^C than long chain *n*-alkanes (Fig. 6). This suggests different biological origins of the short and long chain *n*-alkanes. The terrigenous-to-aquatic ratio (TAR), defined as (*n*-C_27_ + *n*-C_29_ + *n*-C_31_)/(*n*-C_15_ + *n*-C_17_ + *n*-C_19_), has been widely used to quantify terrestrial versus aquatic source contributions in sedimentary organic matter (e.g., refs^53–56^). The TAR values range between 0.07 and 0.75 and average 0.27 ± 0.20 (Fig. 5a). The upper unit has TAR values (0.43 ± 0.23) that are significantly higher than the lower unit (0.16 ± 0.07) (Mann-Whitney U test: *P* = 0.005).

A range of polycyclic aromatic hydrocarbons (PAHs) were identified, and the terrestrial plant-derived compounds are presented in Figs 5b, Supplementary Fig. S2. Retene, perylene, 1,7-dimethylphenanthrene, 1,2,5,6-tetramethylnaphthalene, and 1-methylphenanthren are compounds that potentially indicate the contributions of organic matter from land plants to aquatic sediments^13,57–60^. Although these compounds were detected in all samples, their concentrations do not show a statistically significant difference between the lower and upper unit. The relative concentrations of perylene and the three alkyl PAHs are overall higher in the upper unit, which are 1.1 ± 0.3% and 4.6 ± 0.7% relative to total PAHs, respectively, than those in the lower unit, which are 0.3 ± 0.4 and 4.5 ± 0.4%, respectively (Fig. 5b). The relative concentrations of retene are overall stable in both the upper (0.9 ± 0.3%) and lower (0.9 ± 0.2%) units. PAHs that have been previously used to reconstruct land plant burning and forest fires in geological history were detected in all samples. These include pyrene, benzo(a)pyrene, benzo(e)pyrene and coronene (Supplementary Fig. S2)^13,61^. The relative concentrations of these combustion-derived PAHs show no significant change from the lower to the upper unit (Mann-Whitney U test: *P* = 1.000).

### Inorganic geochemical characteristics of the chattanooga shale

Based on X-ray fluorescence (XRF) analyses, the samples contain SiO_2_ (64.8 ± 12.8 wt.%), Al_2_O_3_ (11.3 ± 1.2 wt.%), K_2_O (2.9 ± 0.9 wt.%), Na_2_O (0.6 ± 0.2 wt.%) and CaO (0.5 ± 0.5 wt.%). The Chemical Index of Alteration (CIA = [Al_2_O_3_/(Al_2_O_3_ + Na_2_O + CaO + K_2_O)] × 100) and the Chemical Proxy of Alteration (CPA = [Al_2_O_3_/(Al_2_O_3_ + Na_2_O)] × 100) were calculated as previously described^62,63^. These two proxies provide a quantitative measure for silicate rock and soil weathering, since alumina becomes increasingly enriched throughout the weathering process and sodium, calcium and potassium are more preferentially removed^62,63^. CIA values differ significantly between the two stratigraphic units (Mann-Whitney U test: *P* = 0.008), showing a lower mean value (67.5 ± 5.0) in the lower unit than the upper unit (73.1 ± 4.3) (Fig. 4). Similarly, CPA values show a significantly lower value (91.6 ± 1.1) in the lower unit than in the upper unit (93.3 ± 1.7, Mann-Whitney U test *P* = 0.006) (Fig. 4).

X-ray diffraction (XRD) analyses show that quartz and clay minerals are the main components in the Chattanooga Shale section. Quartz is the most abundant mineral; it varies in a large range, however, from 6.6% to 90.5% (57.9 ± 20.2%). Clay minerals are the next abundant, ranging from 12.0% to 92.2% (36.3 ± 21.6%), and chlorite and illite are the two most abundant minerals. The quartz-to-clay ratio increases significantly from 1.7 ± 1.0 in the lower unit to 3.6 ± 2.4 in the upper unit (Mann-Whitney U test: *P* = 0.189). This pattern is in agreement with the XRF data that show an increasing trend in the ratios of SiO_2_ to Al_2_O_3_, from 5.1 ± 1.1 in the lower unit to 7.2 ± 1.3 in the upper unit (Mann-Whitney U test: *P* = 0.001) (Fig. 4). Crystal forms examined under SEM show that the quartz component is primarily made up of detrital grains (Supplementary Fig. S9), instead of originating from silica-filled cysts of green algae *Tasmanites,* which instead appear as flattened organic spheres lacking early quartz cement. Therefore, the variation in SiO_2_ primarily reflects changes in the relative amount of terrigenous materials. On the other hand, Al-normalized concentrations of Ti and Zr, Ti/Al and Zr/Al ratios in the lower unit are (0.07 ± 0.01 and (2.19 ± 0.27) × 10^−3^ respectively) significantly lower relative to the values for the upper unit (0.09 ± 0.01 and (3.28 ± 0.11) × 10^−3^, respectively; Mann-Whitney U test: *P* ≤ 0.003) (Fig. 4). Ti and Zr are thought to be contributed by high-density minerals such as zircon, rutile, sphene, and ilmenite^64^. This increasing trend in the studied section suggests a change in mineral assemblages during the deposition of the Chattanooga Shale.

## Discussion

Although black shales in eastern North America may not preserve abundant macrofossils of Devonian trees and shrubs, our geochemical and microscopic data show that the signatures of afforestation can be identified in offshore environments. Here, we present three main lines of geochemical evidence that demonstrate land plants contributed organic and inorganic terrestrial material to offshore environments observed throughout the deposition of the Chattanooga Shale. First, plant body parts and combustion residue (e.g., wood pieces, spores, vitrinite, inertinite) throughout the entire Alabama section, provide the most direct, visible evidence of the land plant contribution to the Chattanooga Shale (Figs 3, Supplementary Figs S7, S8). Vitrinite is thought to be derived from wood tissues^41,65,66^, and inertinite represents highly oxidized materials generated from slow oxidation of organic matter or rapid oxidation during wildfires^49,67–69^. In the studied section, vitrinite and inertinite show an overall increasing trend from the lower to upper unit (Fig. 4). Similarly, Rimmer *et al*. (ref.^48^) also reported this pattern of increases in inertinite from the uppermost Devonian terrestrial and marine rocks (including black shales) in the northern Appalachian Basin, and it was interpreted to be a result of an increasing occurrence of wildfires.

The second line of geochemical evidence is based on biomarkers including PAHs and normal alkanes. PAH compounds including pyrene, benzo(a)pyrene, benzo(e)pyrene and coronene (Fig. 5b) possibly indicate land plant burning. These PAH compounds have been used to indicate wildfire events throughout the Phanerozoic from a diverse type of rocks and sediments, including Devonian marine sedimentary rocks^13,46,52,70^. For example, benzo(a)pyrene, benzo(e)pyrene, pyrene and coronene co-occurring with inertinite were reported from Upper Devonian marine rocks in Poland as the evidence of paleo-wildfires in the eastern Avalonia^46^. In addition to combustion-related PAHs, a range of compounds that may indicate the occurrence of terrestrial plant material are present in our samples, including retene, perylene, long chain *n*-alkanes, 1,7-dimethylphenanthrene, 1,2,5,6-tertramethylnaphathalene, and 1-methylphenanthrene (Fig. 5). Retene is structurally similar to abietane that is derived from the conifer biomarker abietic acid. Although the oldest macrofossil record of conifers was reported from the Late Carboniferous^71^, the earliest tracheophytes may also produce the conifer biomarkers^60^. The occurrence of retene in ancient rocks has been considered to be strong evidence for the contributions of early terrestrial higher plant^58,72–74^. Perylene is believed to originate from the activity of wood-degrading fungi^59,75^. It is frequently found in sediments and crude oils dating back to the Mesozoic but appears to be largely absent in marine sediments lacking terrestrial input and samples deposited before the rise of vascular plants^59^. Previous studies have used perylene in Devonian marine formations to reflect organic matter contributions from terrestrial higher plants (e.g., refs^13,60,76^). In our samples, retene and perylene were detected throughout the studied section, supporting the presence of organic matter from terrestrial higher plants to the Chattanooga Shale of Alabama (Fig. 5b).

Other biomarkers that are less source-specific but may also indicate terrestrial plants, were also evaluated. Traditionally, short chain (C_15_–C_19_) *n*-alkanes in aquatic sediments have been used to represent contributions from algae and microorganisms^77–79^, whereas long chain *n*-alkanes (≥*n*-C_27_) are thought to originate primarily from terrestrial vascular plants^80,81^. Compound-specific stable carbon isotopes of *n*-alkanes can further differentiate the biological sources of short versus long chain *n*-alkanes^50,82–84^. Based on this assumption, TAR was applied to represent organic matter contributions of land plants relative to marine microorganisms, and it shows an increasing trend from the lower to the upper unit in the Chattanooga Shale (Fig. 5a). The assumption that long chain and short chain *n*-alkanes originate from different biological origins is supported by the observation that the δ^13^C values of long chain *n*-alkanes are more depleted than the short chain counterparts (Fig. 6). It also needs to be noted that although long chain *n*-alkanes are among the most widely utilized biomarkers for terrestrial higher land plants^80,85,86^, mosses and the non-marine microalgae, *Botryococcus braunii*, also produce these compounds^87,88^. Despite this source ambiguity, the TAR ratios in our samples show a strong covariation with the abundance of inertinite and vitrinite (Pearson’s *r* = 0.702, *P* = 0.004), supporting the idea that TAR can be used as a proxy of variation in land plant input to the Chattanooga Shale. Additionally, several PAH compounds, 1,7-dimethylphenanthrene, 1,2,5,6-tertramethylnaphathalene, and 1-methylphenanthrene, are present in our samples (Figs 5, Supplementary Fig. S2). These compounds are generally not considered as source-specific terrestrial plant biomarkers as they may be derived from aromatization of organic matter of various biological origins. Terpenoids structures that are prevalent among land plants is one likely source^89^, and these compounds have been previously used to indicate land plant input into marine sediments (e.g., refs^58,60,90^).

The third line of evidence lies in inorganic geochemical proxies, which show that continental weathering became more intense during the deposition of the Chattanooga Shale. SiO_2_/Al_2_O_3_, CIA, and CPA all show an increasing trend from the lower to upper unit (Fig. 4). SiO_2_/Al_2_O_3_ is a useful indicator for changes of detrital input into marine environments^91,92^. CIA and CPA calculated from marine sediments have been widely used to evaluate the chemical weathering intensity of source areas and rocks (e.g., refs^93–96^). Both CIA and CPA are positively correlated with SiO_2_/Al_2_O_3_ (CIA vs. SiO_2_/Al_2_O_3_: Pearson’s *r* = 0.662, *P* = 0.001; CPA vs. SiO_2_/Al_2_O_3_: Pearson’s *r* = 0.621, *P* = 004), and their increasing trends indicate an increase in terrigenous quartz input accompanied by intensification of weathering on land during the deposition of the Chattanooga Shale. Correspondingly, TOC concentrations in the upper unit are lower, probably reflecting an increasing dilution of *in situ* produced marine organic material caused by increasing amount of continental clastic material. It needs to be acknowledged that marine productivity declines can also lead to the TOC concentration reduction, but this interpretation does not agree with the higher δ^13^C values and total phosphorous in the upper unit (Figs 4 and 5). Additionally, the upward increases in Ti/Al and Zr/Al suggest that heavier, coarse minerals were deposited over time. This reflects a stronger force of mobilizing allocthonous minerals^97,98^ and further confirms the increased contribution of terrigenous sediments. The Ti/Al and Zr/Al ratios have also been used in other Upper Devonian marine sedimentary sequences to indicate the relative contribution of heavy minerals and the strength of material transportation from land to sea^52,92,99–101^.

**Figure 7:**
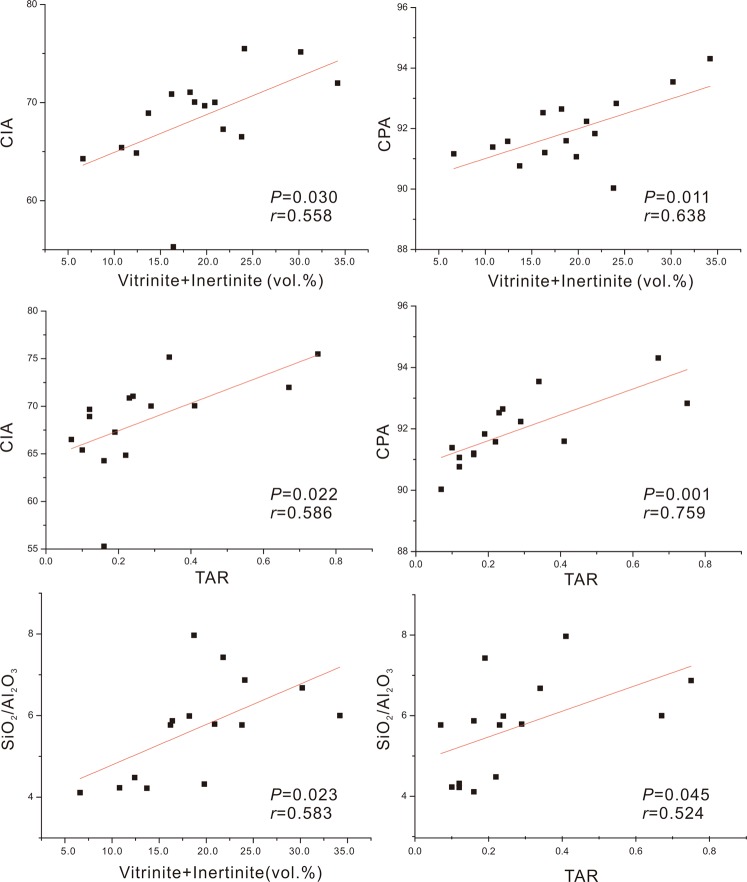
Cross plots of terrestrial plant proxies versus continental weathering proxies from the Upper Devonian Chattanooga Shale in northeastern Alabama. Pearson’s *P* and *r* values are presented. Red lines denote linear regression lines. The positive significant correlations between the indicators for plant inputs and weathering intensity suggest that early land plants likely intensified continental weathering.

The enhancement in continental weathering during the deposition of the Chattanooga Shale may be caused by a climatic shift to warmer and wetter conditions, yet this explanation contradict previous data suggesting that the early and middle Famennian climate become cooler and drier globally and near the study area^25,102^. More likely, the intensified continental weathering is due to land plant invasion onto unvegetated, barren lands. The early development and invasion of rooted land plants to barren lands have been suggested to accelerate physical and chemical weathering of bedrocks through the Devonian^4,103^. The roots of land plants evolved from being small (1–3 mm in diameter and up to 30 cm long), having limited geochemical effects on soils during the Early Devonian^104–107^, to being large (>2.5 cm in diameter), deep (reaching >1 m in depth), and effective in breaking down rocks during the Late Devonian^4,103,104,108^. Our data show significant positive correlations between the proxies of terrestrial plant abundance (TAR, vitrinite and inertinite) and the proxies of continental input and weathering (SiO_2_/Al_2_O_3_, CIA and CPA) (Fig. 7), providing further evidence supporting the interrelated connections among land plants, continental weathering and soil development. The combustion and land plant related PAH compounds show a more scattered pattern (Fig. 5) and do not correlate significantly with the weathering proxies, but their low concentrations make reliable quantifications difficult. Nevertheless, their occurrences throughout the studied section strongly support that land plants were widespread in the southern Acadian land during the Late Devonian.

Combining geochemical analyses, including multiple organic and inorganic geochemical proxies, our data provide the first evidence of afforestation on the southern Acadian land and the associated changes in land-ocean biogeochemical linkages during the Famennian. The occurrences of microfossils (wood fragments and spores) and biomarkers indicate that forests were present during the Famennian time on the southern Acadian land – a paleogeographic region and time that is largely absent of plant records based on conventional investigations of fossils. Several proxies (vitrinite and inertinite, TAR, retene, perylene, chemical weathering indices) further demonstrate that terrestrial plants became an increasing source of organic matter that likely intensified continental weathering and better mobilized clastic materials during the deposition of the Gassaway Member of the Chattanooga Shale.

**Figure 8:**
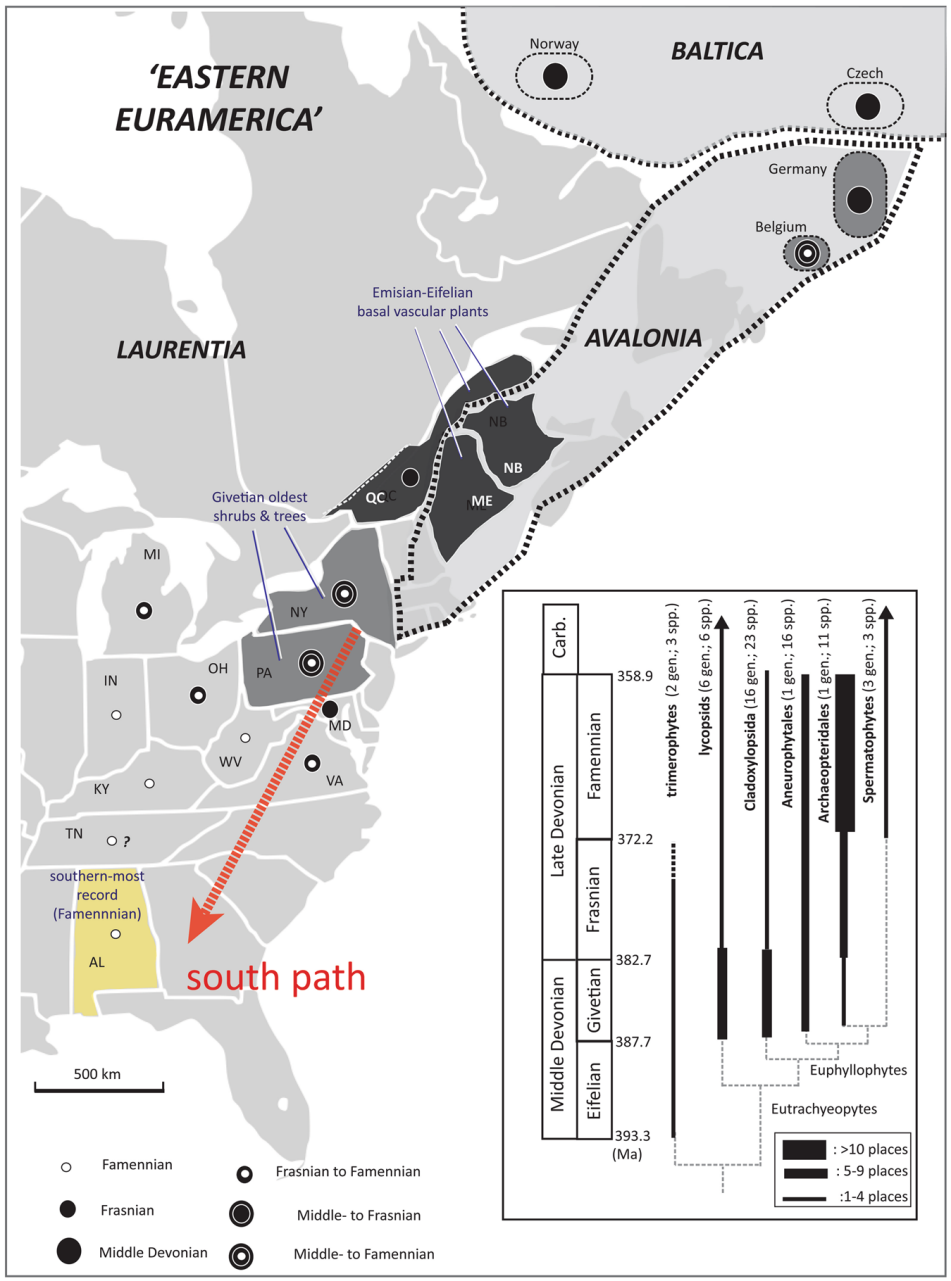
Spatiotemporal dispersal pattern of land forests in southcentral Euramerican landmass during the Devonian. Vascular plant occurrence is summarized by taxa (specific-level), time (Emsian to Famennian ages), and space (state, province, and/or country). Circle size indicates stratigraphic distribution: the older record with a larger symbol (see the legend on left bottom). Three gradients in states/region/country (dark grey, intermediate, and light grey) represent dispersal time-sequence: the older with the dark to the younger with the light color), showing a southern dispersal pattern (the ‘south path’ arrow). A simplified cladogram of higher-level euphyllophytes (selected tree and shrub taxa) show a diversity pattern through the time (i.e., Famennian expansion). Raw data are available in Supplementary Table S2.

Current knowledge of the paleogeographic distribution of Devonian forests and associated soils is primarily based on the fossil record paleobiogeographic occurences of vascualr siltstone successions (e.g., refs^15,16,28,109–111^) (Supplementary Table S1). Our data from black shales, therefore, make an important addition to the scarce records of paleogeographic occurrences of the early forest and soil formations during the Late Devonian by presenting clear evidence of afforestation and the associated input to marine sediments in a paleogeographic area with no previously known records (i.e., the southern Acadian Orogen). Because upper Devonian black shale units are geographically distributed in a large area from the northernmost to southernmost margins of the Appalachian Basin^30^, they overcome the limitation due to the poor preservation of terrestrial deposits and can place the record of afforestation within a detailed biostratigraphic framework. Although the geochemical and microscopic data do not provide diagnostic characteristics to identify specific plant taxa, accumulated information on biostratigraphic and paleobiogeographic occurences of vascular plant fossils (e.g., refs^28,109,112^) can offer a reasonable clue. In southcentral Euramerica along the Acadian landmass, plants with wood tissues, assigned to first shrubs or trees, appeared and soon diversified during the latest Middle Devonian to the end of the Famennian (Fig. 8 and Supplementary Table S1). Because woody tissue (taller and robust stem, megallophyles, and deeper roots) is thought to be advantageous for adapting to or invading drier and more inland environments, those species are thought to be a major contributor of the earliest forests in this paleogeographic region^29,109^.

To place our geochemical data in the context of the spatiotemporal evolution of Devonian forest, we synthesize the fossil record of vascular plants (Eutracheophytes) based on the published data, museum specimens, and the Paleobiology Database (https://paleobiodb.org/). Figure 8 shows a summary of the generic- and species-level occurrence across time (three Age- or Stage-based intervals) and space (state-province-country), which characterizes the Devonian afforestation and pedogenesis (raw data of the figure available in Supplementary Table S1). Stratigraphically, the oldest trees and shrubs (within euphyllophytes) appeared in the Givetian (later Middle Devonian) strata; only a few basal eutracheophtyes, such as the trimerophytes, are known from Emisian-Eifelian strata of the northern Acadian-southern Caledonian orogens (indicated by states/region/country in dark grey on the map of Fig. 8). Although cladoxylopsids were the most dominant group in the Middle Devonian, aneurophytales eventually took over the niches through the Givetian–Frasnian transition. Paleogeographically, the two main tree-shrub bearing groups (taxa assigned to Cladoxylopsida and the progymnosperm Aneurophytopsida) were restricted in the northern Acadian region by the end of the Frasnian (see the stratigraphic occurrence with a cladogram in Fig. 8 and Supplementary Table S2). However, through the Famennian Age, the single taxon of Archaeopteridopsida, *Archaeopteris*–*Callixylon*, had spread from 2 to 12 states/provinces. This pattern is unlikely to result from differential preservation in space and time because of two reasons. First, plant fossils have been observed in formations composed of various rock types (e.g., sandstone, siltstone, shale, see Supplementary Table S1), suggesting that vascual plant fossil preservation in the Appalachiain Basin is not selective for rock types. Second, we observe temporal changes in plant fossils for a given formation with little change in lithology. For example, Frasnian shales (e.g., those lower members of the Chattanooga Shale, the Fynn Creek Formation) in the Southern Appalachian have no known wood fossils, but Famennian shales of the Chattanooga Shale contain some well-preserved large tree fossils in central Tennessee (i.e., in the Gassaway Member) (Supplementary Fig. S10). Rather than reflecting preservation differences, these stratigraphic and paleogeographic data reveal two important trends of floral turnover that occurred on a large scale through the Frasnian–Famennian transition near the southcentral Euramerican landmass. First, the global floral turnover had progressed southward (see the red arrow of ‘south path’ on Fig. 8). Second, this global southward dispersal had progressed in a relatively short time during the Frasnian–Famennian transition. Our geochemical data provide the first strong evidence for the southern end of this southward path. We further hypothesize that this transition could have extended further into the southern American landmass of Gondwana as part of global-scale afforestation, progressing from north to south during the Famennian (e.g., refs^18,113^) especially if the physical landbridge between Euramerica and Gondwana existed by the end of the Devonian^114^. The widespread, yet under-utilized, unfossiliferous Devonian black shales may hold the key to test this hypothesis by filling temporal and spatial gaps in the global path of afforestation and pedogenesis.

## Material and Methods

### Samples

Rock samples were collected from 45 layers at a 25-cm interval, which covered all identified or visible lithological changes in the Chattanooga Shale section (Fig. 2). Weathered rocks (i.e., generally turning to light gray) were avoided for sampling, but freshly exposed rocks (i.e., darker color) were chosen. Prior to geochemical analyses, samples were washed in sonicating baths sequentially using ultra-pure carbon-free water, dichloromethane, and hexane.

### X-ray diffraction (XRD), X-ray fluorescence (XRF) and scanning electron microscope (SEM)

Fifteen samples collected at intervals of 25–50 cm were selected for X-ray diffraction analysis on a Bruker D8 Advance XRD. Mineral identification was based on diffraction patterns using the DIFFRAC^PLUS^EVA 4.0 library (Bruker AXS), and the abundances of different mineral components were determined using the Rietveld Method^115^.

Total element contents were determined by X-ray fluorescence spectrometry (Philips PANalytical PW2424, Netherland) at the ALS Chemex Lab, Ltd (Guangzhou, China). Before the analysis, the powdered samples were dissolved using lithium metaborate mixed with lithium nitrate and heated at 1050 °C for an hour. The mixtures were then transferred into a platinum mould and analyzed by XRF spectrometry.

Selected samples were fixed on stubs and coated with carbon for further examination of minerals and microfossils using a JOEL SEM (JSM-6010PLUS/LA). The SEM magnification was set to 500X to 3500X, depending on the size of the particles. The elemental composition of the samples was analyzed on a JEOL 7000 FE SEM equipped with EDX at the Central Analytical Facility, The University of Alabama.

### Trace element

Measurements of trace elements were made using a PerkinElmer Elan9000 element inductively coupled plasma mass spectrometry (ICP-MS) at the ALS Chemex Lab, Ltd (Guangzhou, China). Ground samples were prepared using a four-acid (HF, HNO_3_, HClO_4_, HCl) digestion method^116^. Analytical precision for all elements is better than 7%, and accuracy was evaluated relative to international reference materials, including GBM398-4c, GBM908-10, MRGeo08 and OGGeo08.

### Organic petrography

Samples at an interval of 50 cm were selected for organic petrography analysis. 10 g of each sample was demineralized using cold 10% HCl for 24 h and then cold 48% HF for 48 h. The samples were then treated with hot Schultz’s solution and sodium hydroxide, followed by a water rinse until a neutral pH was achieved. The residues of the samples were embedded in epoxy resin, polished, and observed using reflectance microscopy under a Nikon Microphot microscope. The samples were examined under immersion oil using a ×40 objective lens, and the abundances of vitrinite and inertinite particles were point-counted (500 points). All samples were analyzed in duplicate and mean values of the results are reported in this study.

### TOC and stable carbon isotope of TOC

Samples at intervals of 25–100 cm were analyzed for total organic carbon (TOC) and stable isotopes of total organic carbon (δ^13^C). Samples were ground into 100–200 mesh powder, and approximately 10 mg of powdered samples were placed into tin capsules. Samples were treated with 5% sulfurous acid to remove carbonate and then dried overnight in an oven at 50 °C. The samples were analyzed on a Micro Cube elemental analyzer (Elementar Analysensysteme GmbH, Hanau, Germany) interfaced to a PDZ Europa 20–20 isotope ratio mass spectrometer (The Sercon Ltd., Cheshire, UK) at The University of California Davis Stable Isotope Facility (California, USA). The analytical precision was <0.2‰ based on internal standards (including nylon, bovine liver, peach leaves, and glutamic acid) calibrated against NIST Standard Reference Materials (USGS-40, USGS-41). Our data were reported as δ^13^C values (‰) relative to V-PDB.

### Biomarker quantification and compound-specific stable carbon isotope

Samples at an interval of 25–100 cm were selected for biomarker analysis at the Organic Geochemistry Laboratory, Department of Geological Sciences, University of Alabama. Duplicate samples were analyzed in every five samples, and solvent blanks were taken through the whole procedure for each run. Approximately 5 g powdered samples were ultrasonically extracted (20 minutes) three times with a mixture of 18 mL dichloromethane (DCM) and 2 mL methanol. Blanks (i.e., only the solvent mixture) were analyzed every 5 samples. In order to remove sulfur, short copper turnings were added to the extracts during the extraction process (20 °C) and overnight storage (−20 °C). The extracts were then concentrated to a volume of ca. 1 mL with a gentle ultrahigh purity (UHP) nitrogen stream using a Zymark Turbo Vap LV Evaporator, and the concentrates were then transferred into GC vials. The extracts were further blown dry gently, diluted with 300–350 μL of hexane, and run on an Agilent 7890B gas chromatograph interfaced with an Agilent 5977 A mass selective detector (MSD). The MSD was operated at a full scanning mode in the mass range of m/z 50–700 at 2.3 scans per second at ionizing electron energy of 70 eV. A fused silica capillary column (Agilent Technologies: 30 m × 0.32 mm, DB-5, 0.25 μm) was used with helium as the carrier gas at a rate of 0.9 mL/min. Sample injection was operated in a pulsed splitless mode at 320 °C. The oven temperature was set at 60 °C, held for 1 minute, and increased at a rate of 6 °C/min to 325 °C, held for 20 minutes. External standards are a mixture of C_7_–C_40_ saturated *n*-alkanes (Sigma Aldrich 49453-U, St. Louis, Missouri) and a PM-610 PAH (Ultra Scientific, North Kingstown, Rhode Island) for quantifying aliphatic and aromatic compounds, respectively. The concentration was calculated using a five-point, peak area vs. concentration calibration curve constructed from standard mixtures with known concentrations (concentration from 0.1 to 20 ng/μL). Compound concentrations were reported in values normalized to TOC contents (μg/g TOC) or the relative percentages. Selected samples were separated into aliphatic and aromatic fractions after the precipitation of asphaltenes. The de-asphalted extracts were then separated into saturate, aromatic, and polar fractions using hexane, benzene, and methanol respectively.

For compound-specific carbon isotopes of normal alkanes, saturated hydrocarbon fractions were further separated into *n*-alkanes and branched/cyclic alkanes by urea adduction^117^. The δ^13^C values of *n*-alkanes were measured in duplicate on a Thermo Fisher Trace GC Ultra coupled with a Thermo Fisher MAT-253 mass spectrometer. The GC was fitted with a 60 m × 0.32 mm i.d. A DB-1MS fused silica capillary column with a film thickness of 0.25 μm leading directly into the combustion furnace was used. The GC oven temperature was programmed from 50 °C (1 min) at 1.5 °C/min to 125 °C, then increased to 300 °C at 5 °C/min, and finally held at 300 °C for 30 min. Helium was used as the carrier gas. The isotopic values were calibrated against the reference gas and were reported in the usual “del” notation relative to VPDB. The precision of the measurements was typically <0.5‰. The accuracy of the instrument was evaluated two to three times daily via analyzing a mixture of *n*-alkanes with known δ^13^C values acquired from Indiana University, USA.

## Supplementary information

Supplementary material for Geochemical Evidence of the First Forestation in the Southernmost Euramerica from Upper Devonian (Famennian) Black Shales
