# Supplementary material for: Geochemical Evidence of First Forestation in the Southernmost Euramerica from Upper Devonian (Famennian) Black Shales
Source: Sci Rep. 2019 May 20;9:7581. doi: 10.1038/s41598-019-43993-y (PMC6527553; doi:10.1038/s41598-019-43993-y)
Supplement: Supplementary file 1 — Supplementary material for Geochemical Evidence of the First Forestation in the Southernmost Euramerica from Upper Devonian (Famennian) Black Shales [file 41598_2019_43993_MOESM1_ESM.pdf]

Supplementary material for

Geochemical Evidence of the First Forestation in the Southernmost Euramerica from Upper Devonian (Famennian) Black Shales

Man Lu[1], YueHan Lu[1,8,*], Takehito Ikejiri[1,2], Nicholas Hogancamp[3], Yongge Sun[4], Qihang Wu[5], Richard Carroll[6], Ibrahim Cemen[1], and Jack Pashin[7]

[1] Department of Geological Sciences, Alabama Water Institute, University of Alabama, Tuscaloosa, AL 35485, USA.

[2] Alabama Museum of Natural History, University of Alabama, Tuscaloosa, AL 35485, USA.

[3] Hess Corporation, 1501 McKinney Street, Houston, Texas 77010, USA.

[4] Department of Earth Science, Zhejiang University, Hangzhou 310058, China.

[5] Key Laboratory of Water Quality and Conservation of the Pearl River Delta, Ministry of Education, Institute of Environmental Research at Greater Bay, Guangzhou University, Guangzhou 510006, China.

[6] Energy Investigation Program, Geological Survey of Alabama, Tuscaloosa, AL 35401, USA.

[7] Boone Pickens School of Geology, Oklahoma State University, Stillwater, OK 74078, USA.

[8] Academy for Advanced Interdisciplinary Studies, Southern University of Science and Technology, Shenzhen, Guangdong 518055, China. *Corresponding author, yuehan.lu@ua.edu

Non-plant Lipid Biomarkers

Notes on branched alkanes: Monomethylalkanes (MMAs) were identified in the range of C_{12}–C_{20}, with the dominance of 7-, 5-, 4-, 2- and 3-methyl- isomers (Fig. S1). These compounds are generally thought to represent the inputs of bacteria[1,2,3,4].

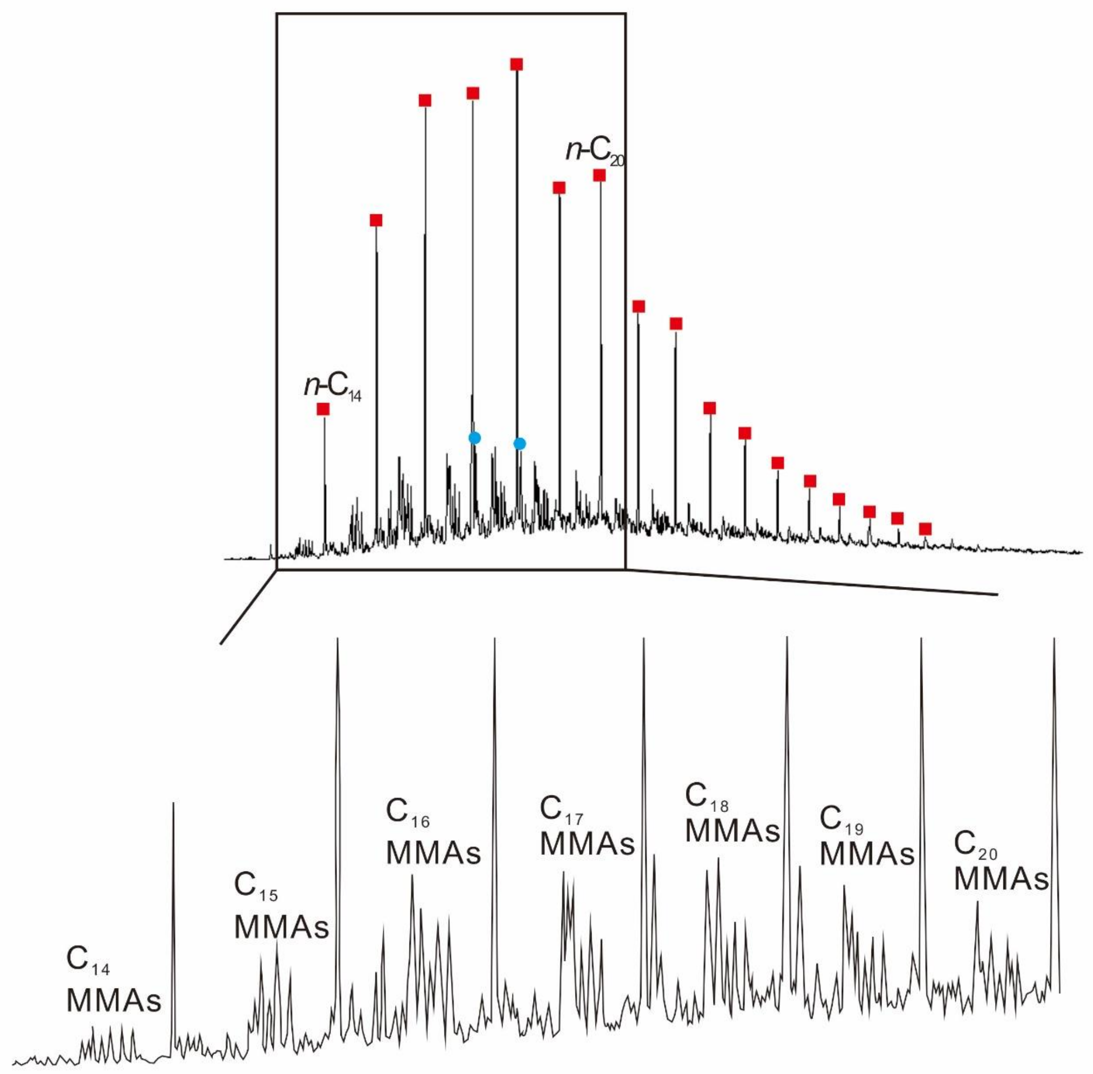

Figure S1. m/z 85 mass chromatogram of a representative sample of the Upper Devonian Chattanooga Shale in northeast Alabama. *Red square* represents normal alkane. *Blue dot* represents isoprenoid.

Notes on PAHs: Abundant constituents include phenantherene and alkylated phenanthrenes, alkylated naphthalenes, chrysene, alkylated chrysene, and perylene (Fig. S2). Among them, 1,7-dimethylphenanthrene, 1,2,5,6-tetramethylnaphthalene, 1-methylphenanthrene, retene and perylene are indicators for contributions from land plants. Pyrene, benzo(a)pyrene, benzo(e)pyrene and coronene are indicators of plant combustion.

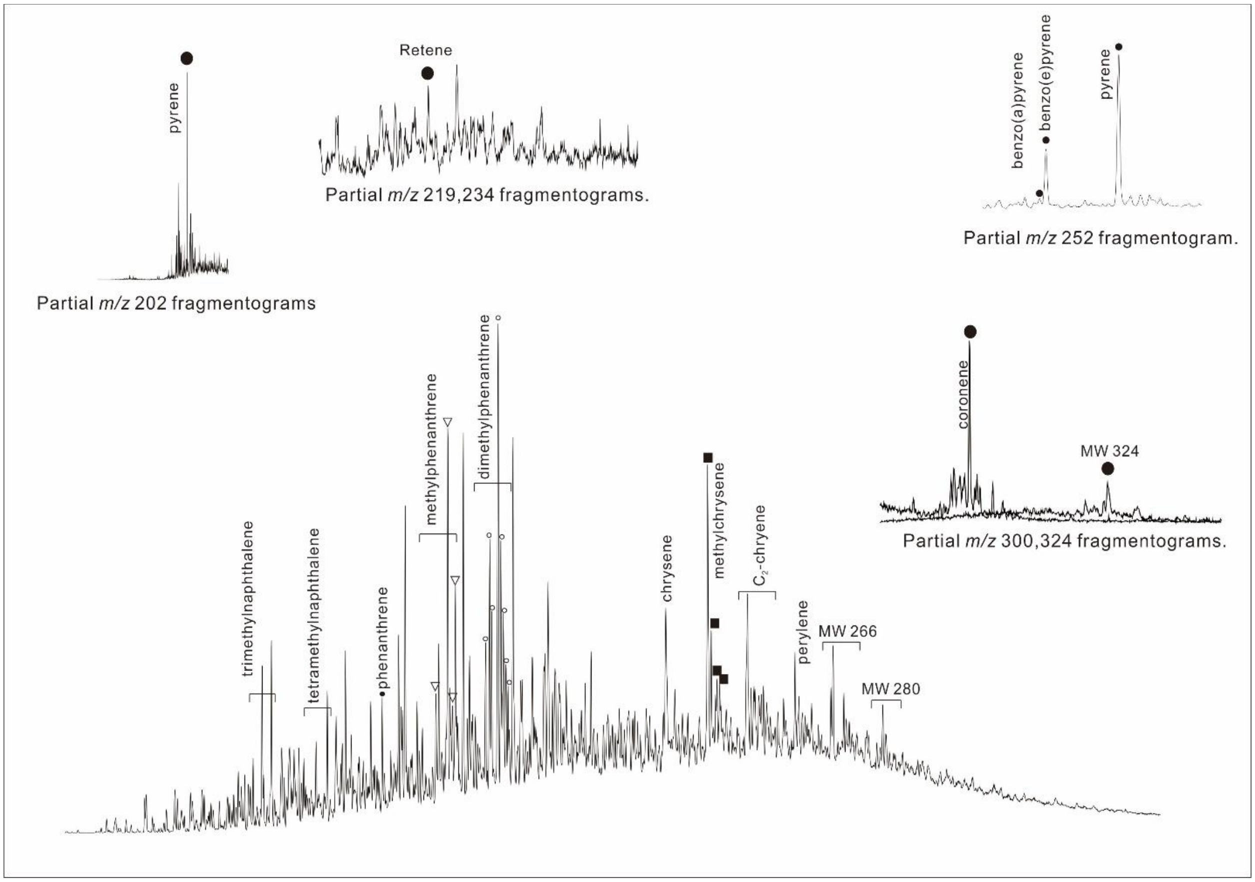

Figure S2. Total ion current (TIC), partial m/z 202, m/z 219/234, m/z 252 and m/z 300/324 chromatograms for one representative sample of the Upper Devonian Chattanooga Shale in northeast Alabama. *Black squares* represent methylchrysene isomers. *Triangles* represent the methylphenanthrene isomers, and *circles* represent dimethylphenanthrene isomers.

Notes on terpenoids: Bicyclic sesquiterpenoids (m/z =123), steranes (m/z=218,217) and hopanes (m/z =191) were also detected (Figs. S3, S4 and S5). These compounds were not quantified because of their low abundances.

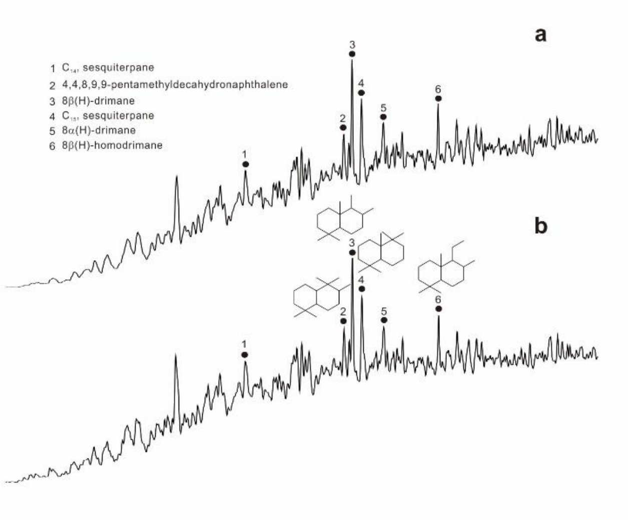

Figure S3. m/z 123 chromatograms showing the distribution of bicyclic sesquiterpenoids for representative samples from the upper unit (**a**) and the low unit (**b**) of the Chattanooga Shale in northeast Alabama.

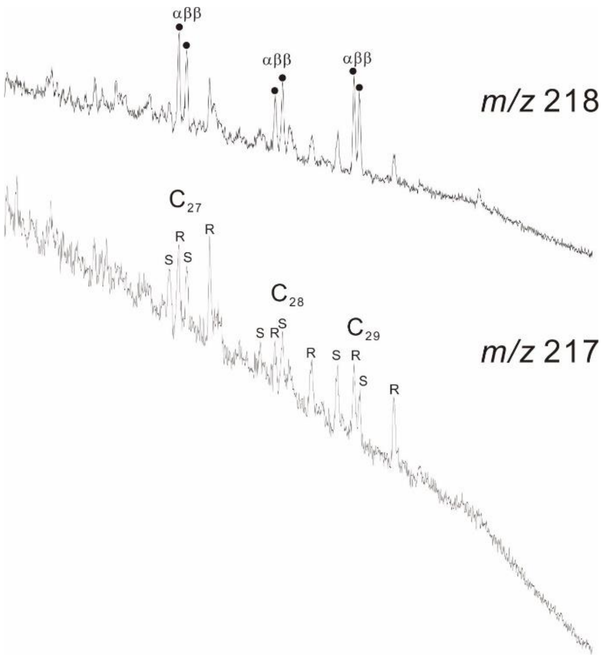

Figure S4. m/z 217 and 218 chromatograms showing the distribution of steranes for a representative sample from the Chattanooga Shale in northeast Alabama.

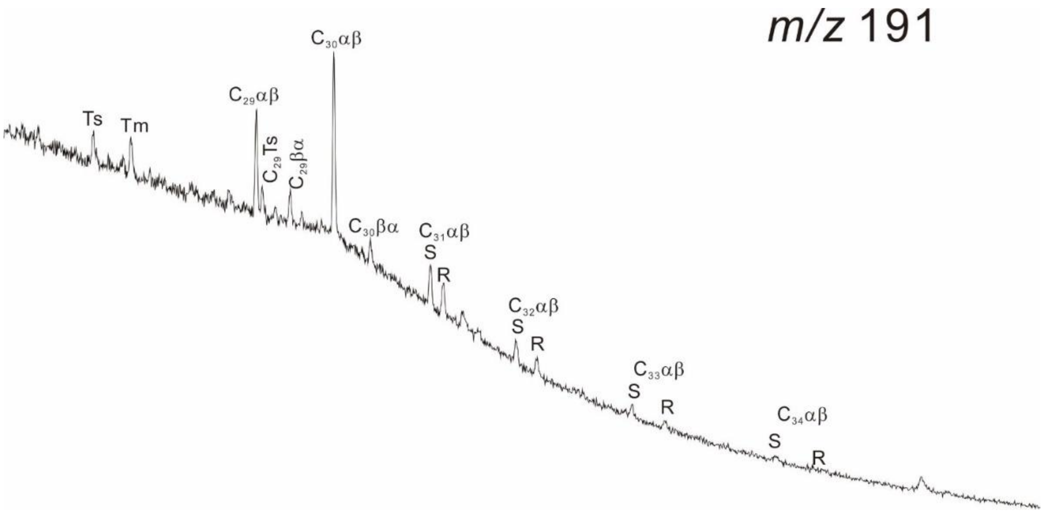

Figure S5. m/z 191 chromatogram showing the distribution of hopanes for a representative sample from the Chattanooga Shale in northeast Alabama.

Microfossils 1: conodonts

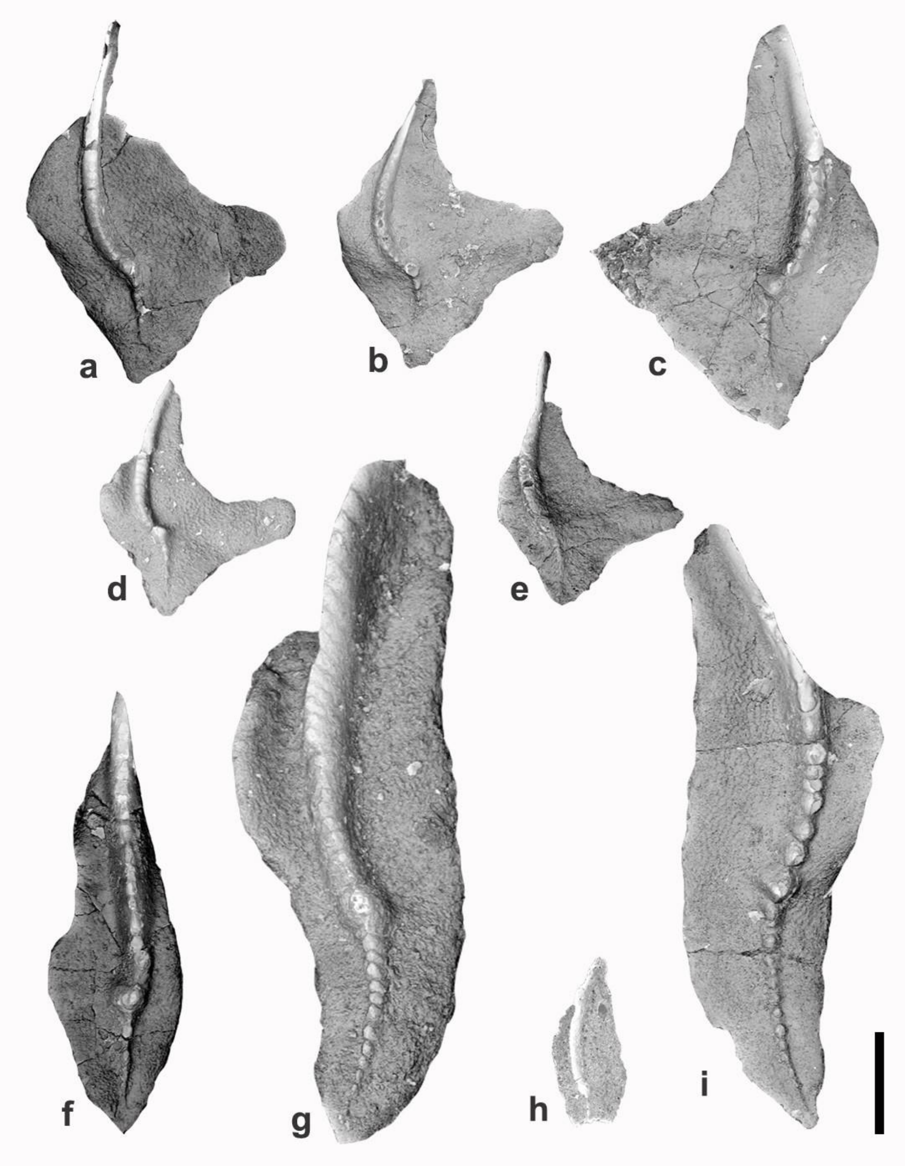

Figure S6. Scanning electron microscope (SEM) images of conodont P_1 element recovered from the Chattanooga Shale in northeast Alabama and occurrence distance above base of shale. Scale bar corresponds to 0.25 mm. a: *Palmatolepis superlobata*, 1.25m; b: *Palmatolepis lobicornis*, 1.75m; c: *Palmatolepis lobicornis*, 1.25m; d: *Palmatoelpis lobicornis*, 1.75m; e: *Palmatolepis superlobata*, 1.00m; f: *Palmatoelpis minuta*, 1.25m; g: *Palmatolepis glabra unca*, 1.70m; h: *Palmatolepis glabra* ssp, 1.00m; i: *Palmatolepis glabra unca*, 1.25m.

Microfossils 2: plants

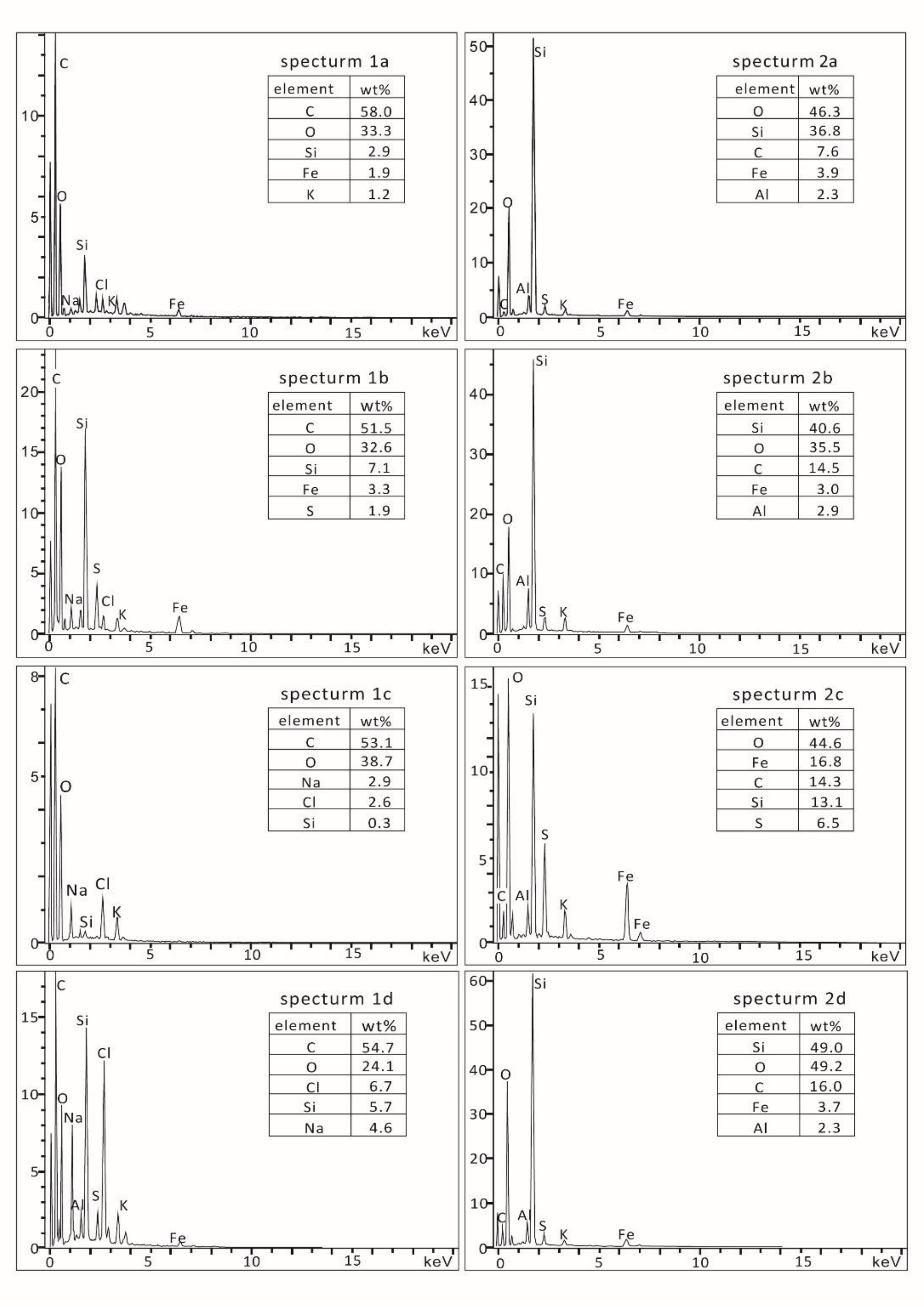

Figure S7. Energy-dispersive X-ray spectroscopy (EDS) spectra of land plant remains from the Upper Devonian Chattanooga Shale in northeastern Alabama. The associated SEM images are in Figure 3 (corresponding to the labels 1a–2d).

Microfossils 3: vitrinite and inertinite

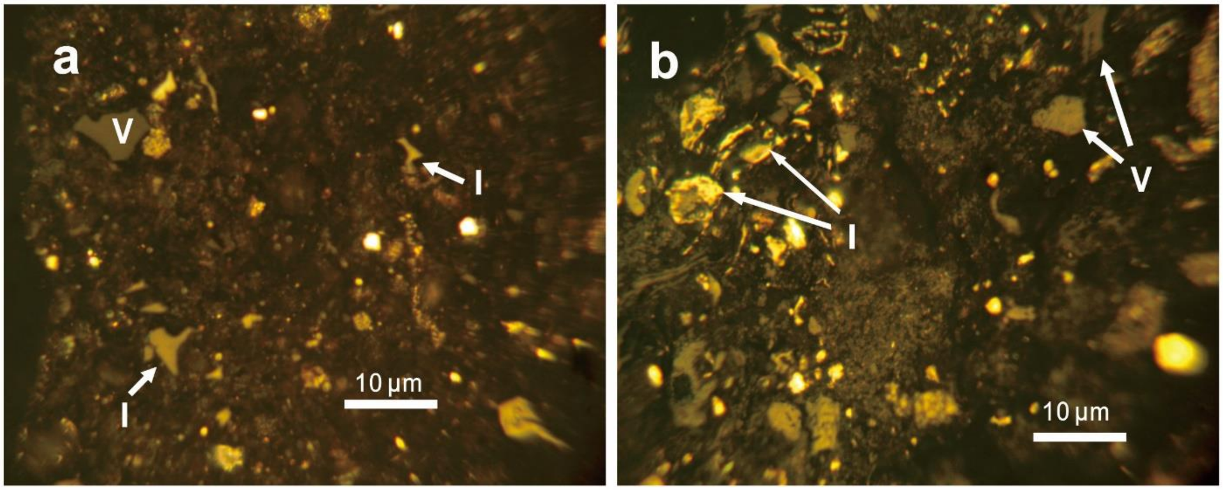

Figure S8. Photomicrographs (white reflected light, oil immersion) of samples from the lower unit (**a**) and the upper unit (**b**) of the Chattanooga Shale in northeast Alabama. **V**=Vitrinite; **I**=Inertinite.

Mineralogy

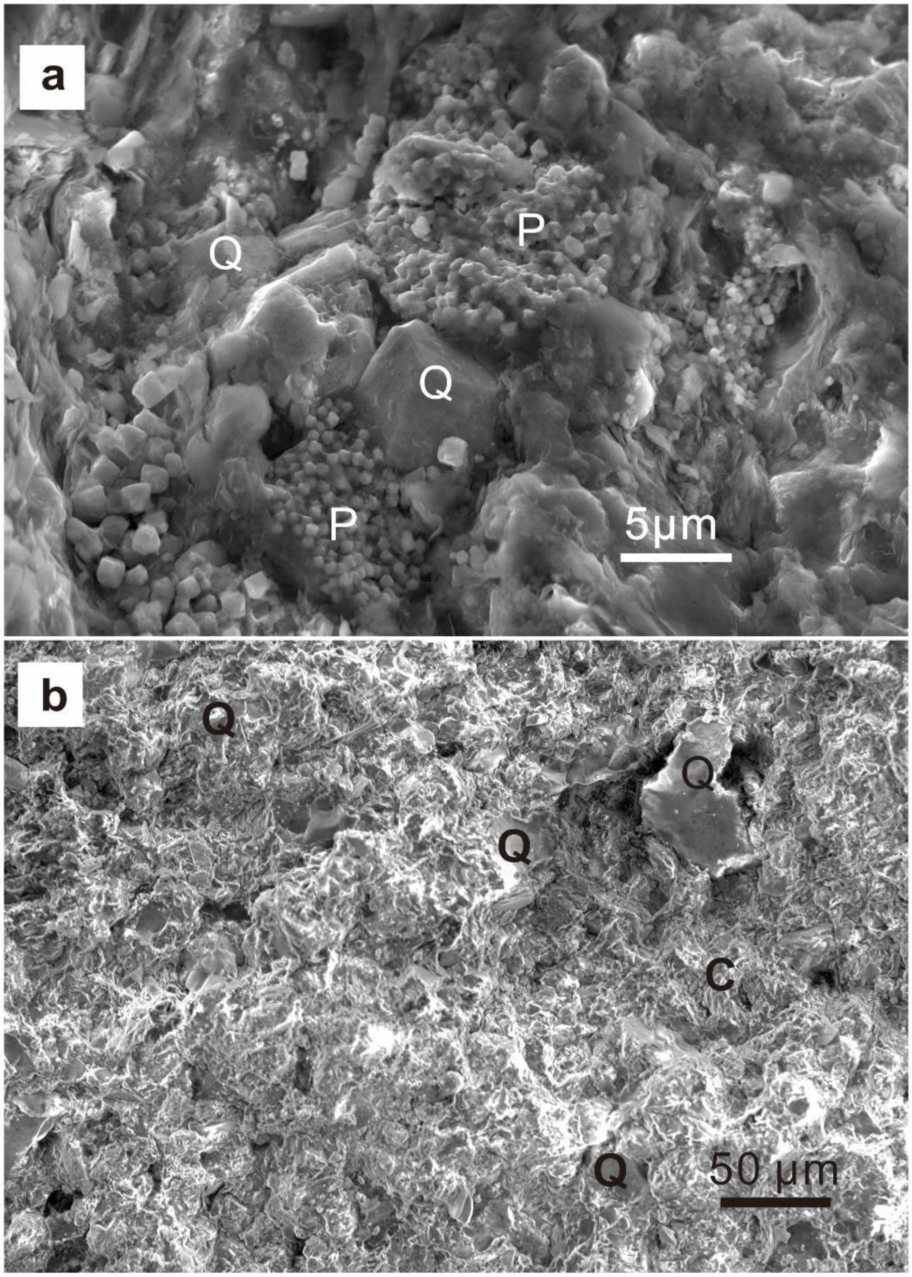

Figure S9. SEM images of mineral crystals from the lower unit (**a**) and the upper unit (**b**) of the Chattanooga Shale in northeast Alabama. **Q**=quartz; **P**= pyrite; **C**= clay minerals. Those quartz crystals are eroded and turned into subangular grains and extensively coated by clay minerals, indicating that quartz in our samples are primarily detrital grains.

Macro-size Plant Fossils

Table S1. A summary of stratigraphic and paleogeographic occurrences of Devonian land forests in the southcentral Euramerica. Vascular plant taxa (Eutracheophytes) with wood tissues, such as trees and shrub are selected. Same data are used in Figure 8. Raw data are listed in Table S2.

A. Generic and specific counts occurred in major taxonomic groups.

	**Lycopsida**		**Cladoxylopsids**		**Anuerophytales**		**Archaeopterids**	
	**genera**	**species**	**genera**	**species**	**genera**	**species**	**genera**	**species**
**Famennian**	3	3	4	4	1	1	1(2)	14
**Frasnian**	1	1	3	3	3	3	1(2)	13
**Middle Devonian**	0	0	9	16	1	4	2	2
**Total**	4	4	13	21	1	6	1(2)	20

	**Spermatophytes**		**Equisetophyta**		**Lepidocarpopsida**	
	**genera**	**species**	**genera**	**species**	**genera**	**species**
**Famennian**	3	3	2	2	1	1
**Frasnian**	0	0	1	2	0	0
**Middle Devonian**	0	0	0	0	0	0
**Total**	3	3	3	4	1	1

B. Generic and specific counts occurred in each region (state/province for US/Canada and country for Europe)

	**Lycopsida**	**Cladoxylopsids**	**Anuerophytales**	**Archaeopterids**
**Famennian**	3	2	2	11
**Frasnian**	1	1	1	7
**Middle Devonian**	5	5	4	1

	**Spermatophytes**	**Equisetophyta**	**Lepidocarpopsida**
**Famennian**	*3*	2	1
**Frasnian**	0	1	0
**Middle Devonian**	0	0	0

Table S2. Occurrence of land plants with wood tissues from Devonian strata of the central Euramerica. Same data are used in Figure 8. Abbreviations for paleogeographic region and state/province; **App Basin**: Appalachian Basin; **NY**: New York; **PA**: Pennsylvania; **WV**: West Virginia. An asterisk symbol (*) in the Formation column indicates a black shale unit.

EMSIAN (Early Devonian)

Higher taxa 1	Higher taxa 2	Genus	Species	Paleogeographic region	Country	State/ Province	Formation	References
Lycopodiophytes	Radiatopses	*Pertica*	*varia*	Avalonia	Canada	Quebec	Battery Point	5
Lycopodiophytes	Radiatopses	*Pertica*	*dalhousii*	Avalonia	Canada	New Brunswick	Campbellton	6, 7;
Lycopodiophytes	Radiatopses	*Trimerophyton*	*robustius*	Avalonia	Canada	Quebec	Battery Point(?)	5
Lycopodiophytes	Radiatopses	*Pertica*	*quadrifaria*	Avalonia	USA	Maine	Trout Valley	7
Lycopodiophytes	Radiatopses	*Pertica*	*quadrifaria*	Avalonia	USA	Maine	Trout Valley	7
Lycopodiophytes	Radiatopses	*Pertica*	*quadrifaria*	Avalonia	USA	Maine	Trout Valley	7

GIVETIAN (Middle Devonian)

Higher taxa 1	Higher taxa 2	Genus	Species	Paleogeographic region	Country	State/ Province	Formation	References
Cladoxylopsida	Pseudosporochnales	*Calamophyton*	*bicephalum*	App Basin	USA	NY	Ashokan	8, 9, 10, 11
Cladoxylopsida	Pseudosporochnales	*Calamophyton*	*bicephalum*	Avalonia	Belgium	Belgium	?	10
Cladoxylopsida	Pseudosporochnales	*Calamophyton*	*primaevum*	Avalonia(?)	Germany	Elberfeld	Honseler Schichten	12, 13
Cladoxylopsida	Pseudosporochnales	*Calamophyton*	*primaevum*	Avalonia	Belgium	Brabant, Ronquieres	Bois de Bordeaux	10
Cladoxylopsida	Pseudosporochnales	*Cladoxylon*	*hueberi*	App Basin	USA	NY	Kiskatom	14
Cladoxylopsida	Pseudosporochnales	*Cladoxylon*	*scoparium*	Avalonia	Germany	?	Honseler Schichten	8
Cladoxylopsida	Pseudosporochnales	*Cladoxylon*	*scoparium*	Avalonia	Germany	Wuppertals	Brandenberg Schichten	8
Cladoxylopsida	Pseudosporochnales	*Cladoxylon*	sp.	App Basin	USA	NY	Kiskatom	15, 16
Cladoxylopsida	Pseudosporochnales	*Wattieza*	*givetiana*	Avalonia	Belgium	Brabant	Fromelennes	17
Cladoxylopsida	Pseudosporochnales	*Wattieza*	sp.	App Basin	USA	NY	Oneonta	18
Cladoxylopsida	Pseudosporochnales	*Rellimia*	*thomsonii*	App Basin	USA	NY	Panther Mountain	19
Cladoxylopsida?		*Eospermatopteris*	*erianus*	App Basin	USA	NY	Moscow	20
Cladoxylopsida?		*Eospermatopteris*	sp.	App Basin	USA	NY	Kiskatom	20
Cladoxylopsida	Pseudosporochnales	*Hyenia*	*banksii*	App Basin	USA	NY	Bellvale Sandstone	21
Cladoxylopsida	Pseudosporochnales	*Hyenia*	*elegans*	Avalonia	Belgium	Brabant	Bois de Bordeaux	22
Cladoxylopsida	Pseudosporochnales	*Hyenia*	*elegans*	Avalonia(?)	Germany	Germany	Honseler Schichten	22
Cladoxylopsida	Pseudosporochnales	*Hyenia*	*vogtii*	Baltica	Norway	Svalbard	Wide Bay	22
Cladoxylopsida	Pseudosporochnales	*Lorophyton*	*goense*	Avalonia	Belgium	?	Pepinster	23
Cladoxylopsida	Pseudosporochnales	*Pseudosporochnus*	*chlupaci*	Baltica	Czech	?	Roblin Schichten; Kacak Schichten; Srbsko-Schichten	24
Cladoxylopsida	Pseudosporochnales	*Pseudosporochnus*	*krejcii*	Avalonia	Belgium	Brabant	Fromelennes	17
Cladoxylopsida	Pseudosporochnales	*Pseudosporochnus*	*krejcii*	Avalonia	Belgium	Ronquieres	Bois de Bordeaux	17
Cladoxylopsida	Pseudosporochnales	*Pseudosporochnus*	*nodosus*	Avalonia	Belgium	Belgium	shale and sandstone	25, 26
Cladoxylopsida	Pseudosporochnales	*Pseudosporochnus*	*nodosus*	Avalonia	Belgium	Ronquieres	Bois de Bordeaux	26

Cladoxylopsida	Pseudosporochnales	*Pseudosporochnus*	sp.	Avalonia	Germany	Hohen Venns	Friiesenrather Schichten	27
Cladoxylopsida	Pseudosporochnales	*Pseudosporochnus*	sp.	App Basin	USA	NY	Kiskatom	14<br>15
Cladoxylopsida	Pseudosporochnales	*Pseudosporochnus*	sp.	App Basin	USA	NY	Ashokan	14<br>15
Cladoxylopsida	Pseudosporochnales	*Pseudosporochnus*	*verticilatus*	Baltica	Czech	Bohemia	Roblin Schichten, Kacak Schichten, Srbsko-Schichten	24
Cladoxylopsida	Pseudosporochnales	*Xenocladia*	*medullosina*	App Basin	USA	NY	Tully Pyrite	28
Cladoxylopsida	Pseudosporochnales	*Xenocladia*	*medullosina*	App Basin	USA	NY	Ludlowville	29
Progymnosperm	Aneurophytopsida	*Aneurophyton*	*bohemicum*	Baltica	Czech	Bohemia	Roblin Schichten, Kacak Schichten, Srbsko-Schichten	24
Progymnosperm	Aneurophytopsida	*Aneurophyton*	*bohemicum*	Avalonia	Germany	Elberfeld	Honseler Schichten	24
Progymnosperm	Aneurophytopsida	*Aneurophyton*	*furcatum*	Avalonia	Belgium	Brabant	Fromelennes	17
Progymnosperm	Aneurophytopsida	*Aneurophyton*	*germanicum*	Avalonia	Belgium	Belgium	<u>Evieux?</u>	30
Progymnosperm	Aneurophytopsida	*Aneurophyton*	*germanicum*	Avalonia	Germany	Germany	Honseler Schichten	31<br>32
Progymnosperm	Aneurophytopsida	*Aneurophyton*	*germanicum*	Avalonia	Germany	Wuppertals	Brandenberg Schichten	8
Progymnosperm	Aneurophytopsida	*Aneurophyton*	*germanicum*	Avalonia	Germany		formations at Wuppertal-Elberfeld Locality	33
Progymnosperm	Aneurophytopsida	*Aneurophyton*	*hallii*	App Basin	USA	NY	Ludlowville	28
Progymnosperm	Aneurophytopsida	*Aneurophyton*	*hallii*	App Basin	USA	NY	Tully Pyrite	28
Progymnosperm	Aneurophytopsida	*Aneurophyton*	sp.	App Basin	USA	NY	Moscow	34 (PBD 28414)
Progymnosperm	Archaeopteridopsida	*Archaeopteris*	*obtuse*	App Basin	USA	NY	Bellvale Sandstone	21
Progymnosperm	Archaeopteridopsida	*Callixylon*	*petryi*	App Basin	USA	NY	Sherbure Sandstone	21
Progymnosperm	Archaeopteridopsida	*Callixylon*	sp.	Avalonia	Belgium	Ronquières	Bois de Bordeaux	76
Progymnosperm	Archaeopteridopsida	*Triloboxylon*	*arnoldii*	App Basin	USA	NY	Kiskatom	15

FRASNIAN (Late Devonian)

Higher taxa 1	Higher taxa 2	Genus	Species	Paleogeographic region	Country	State/ Province	Formation	References
Cladoxylopsida	Pseudosporochnales	*Calamophyton*	*primaevum*	Avalonia	Belgium	Brabant, Ronquieres	Bois de Bordeaux	12; 13
Cladoxylopsida	Pseudosporochnales	*Hyenia*	*elegans*	Avalonia	Belgium	Brabant	Bois de Bordeaux	12 13
Cladoxylopsida	Pseudosporochnales	*Pseudosporochnus*	*krejcii*	Avalonia	Belgium	Ronquieres	Bois de Bordeaux	12 13
Cladoxylopsida	Pseudosporochnales	*Pseudosporochnus*	*nodosus*	Avalonia	Belgium	Ronquieres	Bois de Bordeaux	12, 13
Lycopsida	isoetalean	*Lepidosigillaria*	*whitei*	Acadian	USA	NY	?	35
Lycopsida	Protolepidodendrales	*Protolepidodendropsis*	*pulchra*	Baltica	Norway	Salvard	Planteklofta	74
Lycopsida	Protolepidodendrales	*Lepidosigillaria*	*whitei*	Acadian	USA	NY		
Equisetophyta	Sphenopsida	*Calamospora*	*atava*	Acadian	USA	Maryland	Foreknobs	36
Equisetophyta	Sphenopsida	*Calamospora*	*nigrata*	Acadian	USA	Maryland	Foreknobs	36
Cladoxylopsida	Pseudosporochnales	*Cladoxylon*	*dawsoni*	Acadian	USA	NY	Genundewa Limestone	37, 38
Cladoxylopsida?		*Eospermatopteris*	sp.	Acadian	USA	NY	Oneonta, Stony Clove	20
Cladoxylopsida	Pseudosporochnales	*Rhymokalon*	*trichium*	Acadian	USA	NY	Oneonta	16
Progymnosperm	Aneurophytopsida	*Aneurophyton*	*germanicum*	Acadian	USA	NY	Delaware River Flags	39
Progymnosperm	Aneurophytopsida	*Aneurophyton*	*hallii*	Acadian	USA	NY	Ithaca	34
Progymnosperm	Aneurophytopsida	*Aneurophyton*	*rachides*	Acadian	USA	NY	Oneonta	34
Progymnosperm	Archaeopteridopsida	*Archaeopteris*	*fissilis*	N. Euramerica	Canada	Nunavut	black-gray shale*	34
Progymnosperm	Archaeopteridopsida	*Archaeopteris*	*gaspiensis*	Avalonia	Canada	Quebec	Hugh Miller Cliffs	40
Progymnosperm	Archaeopteridopsida	*Archaeopteris*	*halliana*	Avalonia	Canada	Quebec	Escuminac	40
Progymnosperm	Archaeopteridopsida	*Archaeopteris*	*halliana*	Acadian	USA	NY	Chemung; Katzberg	41, 42
Progymnosperm	Archaeopteridopsida	*Archaeopteris*	*halliana*	Acadian	USA	PA	Chemung	43
Progymnosperm	Archaeopteridopsida	*Archaeopteris*	*hibernica*	Acadian	USA	NY	Katzberg	44, 41
Progymnosperm	Archaeopteridopsida	*Archaeopteris*	*jacksoni*	Avalonia	Canada	Quebec		40
Progymnosperm	Archaeopteridopsida	*Archaeopteris*	*macilenta*	Acadian	USA	NY	Chemung; Katzberg; Oneonta; Stony Clove	41, 43, 45
Progymnosperm	Archaeopteridopsida	*Archaeopteris*	*minor*	Acadian	USA	PA	Chemung	43
Progymnosperm	Archaeopteridopsida	*Archaeopteris*	*obtusa*	N. Euramerica	Canada	Nunavut	black-gray shale	34
Progymnosperm	Archaeopteridopsida	*Archaeopteris*	*obtusa*	Avalonia	Canada	Quebec	Escuminac; Hugh Miller Cliffs	6
Progymnosperm	Archaeopteridopsida	*Archaeopteris*	*obtusa*	Acadian	USA	NY	Katzberg	41
Progymnosperm	Archaeopteridopsida	*Archaeopteris*	*rogersi*	Acadian	USA	PA	Chemung	43
Progymnosperm	Archaeopteridopsida	*Archaeopteris*	*sphenophyllifolia*	Acadian	USA	NY	Delaware River Flags; Katzberg	41
Progymnosperm	Archaeopteridopsida	*Archaeopteris*	sp.	N. Euramerica	Canada	Alberta	Yahatinda	47
Progymnosperm	Archaeopteridopsida	*Archaeopteris*	sp.	Avalonia	Canada	Quebec	Escuminac	40
Progymnosperm	Archaeopteridopsida	*Archaeopteris*	sp.	Acadian	USA	NY	Oneonta	41
Progymnosperm	Archaeopteridopsida	*Archaeopteris*	sp.	Acadian	USA	NY	Enfield; Honesdale; Katsberg; Walton; Stony Clove;	34

Progymnosperm	Archaeopteridopsida	*Archaeopteris*	sp.	Acadian	USA	PA	West Hall (Beers Hill) Chemung; (lower) Pocono	42
Progymnosperm	Archaeopteridopsida	*Callixylon*	*newberryi*	Michigan Basin	USA	Michigan	Antrim Shale	48
Progymnosperm	Archaeopteridopsida	*Callixylon*	*newberryi*	Ohio Basin	USA	Ohio	Olentangy Shale*	49
Progymnosperm	Archaeopteridopsida	*Callixylon*	*petryi*	Acadian	USA	NY	Geneseel Oneonta	38, 41,
Progymnosperm	Archaeopteridopsida	*Callixylon*	*zalesskyi*	Acadian	USA	NY	Katsberg; Oneonta	41, 45
Progymnosperm	Archaeopteridopsida	*Callixylon*	sp.	N. Euramerica	Canada	Nunavut	?	50
Progymnosperm	Archaeopteridopsida	*Callixylon*	sp.	Acadian	USA	NY	Genessee (Genundewa Limestone)	37, 51,
Progymnosperm	Archaeopteridopsida	*Callixylon*	sp.	Acadian	USA	NY	Katzberg	41
Progymnosperm	Archaeopteridopsida	*Callixylon*	sp.	Acadian	USA	NY	Stony Clove	34 (PBD6895)
Progymnosperm	Archaeopteridopsida	*Callixylon*	sp.	Acadian	USA	NY	Oneonta	41
Progymnosperm	Archaeopteridopsida	*Callixylon*	sp.	App Basin	USA	Virginia	Foreknobs	52
Progymnosperm	Archaeopteridopsida	*Callixylon*	sp.	Avalonia	Belgium	Ronquières	Bois de Bordeaux	75
Pinophyta	Pinopsida	*Cordaites*	*angustifolia*	Avalonia	Canada	Quebec	?	40

FAMENNIAN (Late Devonian)

Higher taxa 1	Higher taxa 2	Genus	Species	Paleogeographic region	Country	State/ Province	Formation	References
Equisetophyta	Equisetopsida	*Eviostachya*	*hoegii*	Avalonia	Belgium	Namur	Evieux	53
Equisetophyta	Equisetopsida	*Eviostachya*	sp.	App Basin	USA	WV	Hampshire	54
Equisetophyta	Equisetopsida	*Sphenophyllum*	*subtenerrimum*	Avalonia	Belgium	Liege		55
Equisetophyta	Equisetopsida	*Sphenophyllum*	*subtenerrimum*	App Basin	USA	WV	Hampshire	54
Lycopsida	Lycophytes	*Cyclostigma*	sp.	Acadian	USA	PA		56
Lycopsida	Isoetales	*Lepidodendropsis*	sp.	Acadian	USA	PA	Catskill	57
Lycopsida	Lycopsida	*Otzinachsonia*	*beerboweri*	Acadian	USA	PA	Catskill	58
Isoetophytina	Lepidocarpopsida	*Jurinodendron*	*brevifolium*	Avalonia	Belgium	Namur	Evieux	59
Cladoxylopsida	Pseudosporochnales	*Cladoxylon*	sp.	Illinois Basin	USA	Indiana	New Albany Shale*	60, 61
Cladoxylopsida	Pseudosporochnales	*Hierogramma* (= *Cladoxylon*?)	*jeffreyi*	App Basin	USA	Kentucky	New Albany Shale*; Hampshire	57, 62
Cladoxylopsida	Pseudosporochnales	*Pietzschia*	*polyupsilon*	Illinois Basin	USA	Indiana	New Albany Shale*	60
Cladoxylopsida	Pseudosporochnales	*Pietzschia*	*polyupsilon*	App Basin	USA	Kentucky	New Albany Shale*	60
Cladoxylopsida	Pseudosporochnales	*Polyxylon*	*elegans*	Illinois Basin	USA	Indiana	New Albany Shale*	60
Progymnosperm	Aneurophytopsida	*Aneurophyton*	*olnense*	Avalonia	Belgium	Namur	Evieux	53
Progymnosperm	Aneurophytopsida	*Aneurophyton*	*olnense*	App Basin	USA	WV	Hampshire	54
Progymnosperm	Archaeopteridopsida	*Archaeopteris*	*eastmanii*	App Basin	USA	Kentucky	New Albany Shale*	63
Progymnosperm	Archaeopteridopsida	*Archaeopteris*	*fissilis*	N. Euramerica	Canada	Nunavut	sandstone/shale	37, 51
Progymnosperm	Archaeopteridopsida	*Archaeopteris*	*halliana*	Acadian	USA	NY	Chemung	43
Progymnosperm	Archaeopteridopsida	*Archaeopteris*	*halliana*	Acadian	USA	PA	Chemung	43
Progymnosperm	Archaeopteridopsida	*Archaeopteris*	*halliana*	App Basin	USA	WV	Hampshire	64
Progymnosperm	Archaeopteridopsida	*Archaeopteris*	*latifolia*	Acadian n	USA	PA	Oswayo Sandstone	43
Progymnosperm	Archaeopteridopsida	*Archaeopteris*	*macilenta*	App Basin	USA	WV	Hampshire	54
Progymnosperm	Archaeopteridopsida	*Archaeopteris*	*minor*	Acadian	USA	PA	Chery Ridge Red Shale; Chemung	43
Progymnosperm	Archaeopteridopsida	*Archaeopteris*	*obtusa*	N. Euramerica	Canada	Nunavut	sandstone/shale	50
Progymnosperm	Archaeopteridopsida	*Archaeopteris*	*obusa*	App Basin	USA	WV	Hampshire	54
Progymnosperm	Archaeopteridopsida	*Archaeopteris*	*obusa*	App Basin	USA	Virginia	Hampshire	54
Progymnosperm	Archaeopteridopsida	*Archaeopteris*	*roemeriana*	Avalonia	Belgium	Namur Province	Evieux	65
Progymnosperm	Archaeopteridopsida	*Archaeopteris*	*roemeriana*	Avalonia	Belgium	Namur		65
Progymnosperm	Archaeopteridopsida	*Archaeopteris*	*roemeriana*	Acadian	USA	PA	Pocono	43
Progymnosperm	Archaeopteridopsida	*Archaeopteris*	*rogersi*	Acadian	USA	PA	Chemung	43
Progymnosperm	Archaeopteridopsida	*Archaeopteris*	*sphenophyllifolia*	App Basin	USA	WV	Hampshire	54
Progymnosperm	Archaeopteridopsida	*Archaeopteris*	sp.	Acadian	USA	NY	Cattaraugus;	43, 34 (PBD 29005)

							Conneaut (Oswayo)	
Progymnosperm	Archaeopteridopsida	*Archaeopteris*	sp.	Acadian	USA	PA	Catskill	34
Progymnosperm	Archaeopteridopsida	*Archaeopteris*	sp.	App Basin	USA	WV	Hampshire	66
Progymnosperm	Archaeopteridopsida	*Callixylon*	*brownii*	Illinois Basin	USA	Indiana	New Albany Shale*	60
Progymnosperm	Archaeopteridopsida	*Callixylon*	*brownii*	App Basin	USA	Kentucky	New Albany Shale*	34
Progymnosperm	Archaeopteridopsida	*Callixylon*	*clevelandensis*	Ohio Basin	USA	Ohio	Ohio Black Shale	67
Progymnosperm	Archaeopteridopsida	*Callixylon*	*erianum*	Acadian	USA	NY	Gowanda shale	68
Progymnosperm	Archaeopteridopsida	*Callixylon*	*erianum*	App Basin	USA	WV	Hampshire	54
Progymnosperm	Archaeopteridopsida	*Callixylon*	*newberryi*	Illinois Basin	USA	Indiana	New Albany Shale* (Blackiston)	69
Progymnosperm	Archaeopteridopsida	*Callixylon*	*newberryi*	Illinois Basin	USA	Indiana	New Albany Shale*	60
Progymnosperm	Archaeopteridopsida	*Callixylon*	*trifilievi*	N. Euramerica	Canada	Alberta	Beaverhill Lake	70
Progymnosperm	Archaeopteridopsida	*Callixylon*	sp.	N. Euramerica	Canada	Nunavut	black gray shale	50
Progymnosperm	Archaeopteridopsida	*Callixylon*	sp.	Acadian	USA	PA	Oswayo Sandstone; Pocono; Cuba silt shale (Chadakoin)	43
Progymnosperm	Archaeopteridopsida	*Callixylon*	sp.	App Basin	USA	Virginia	Hampshire	64
Progymnosperm	Archaeopteridopsida	*Callixylon*	sp.	App Basin	USA	WV	Hampshire	54
Progymnosperm	Archaeopteridopsida	*Callixylon*(?)		App Basin	USA	Tennessee	Chattanooga	71
Lycopsida	Lycophytes	*Cyclostigma*		Acadian	USA	PA		56
Gymnospermopsida	spermatophytes	*Aglosperma*	*quadripartita*	Acadian	USA	PA	Catskill	72
Gymnospermopsida	spermatophytes	*Aglosperma*	*bertrandii*	Avalonia	Belgium	?		72
Gymnospermopsida	spermatophytes	*Aporoxylon*	*?*	Avalonia(?)	Germany	Thuringia		73
Gymnospermopsida	spermatophytes	*Araucarites*	*?*	Acadian	USA	NY	Albany Shale*	73

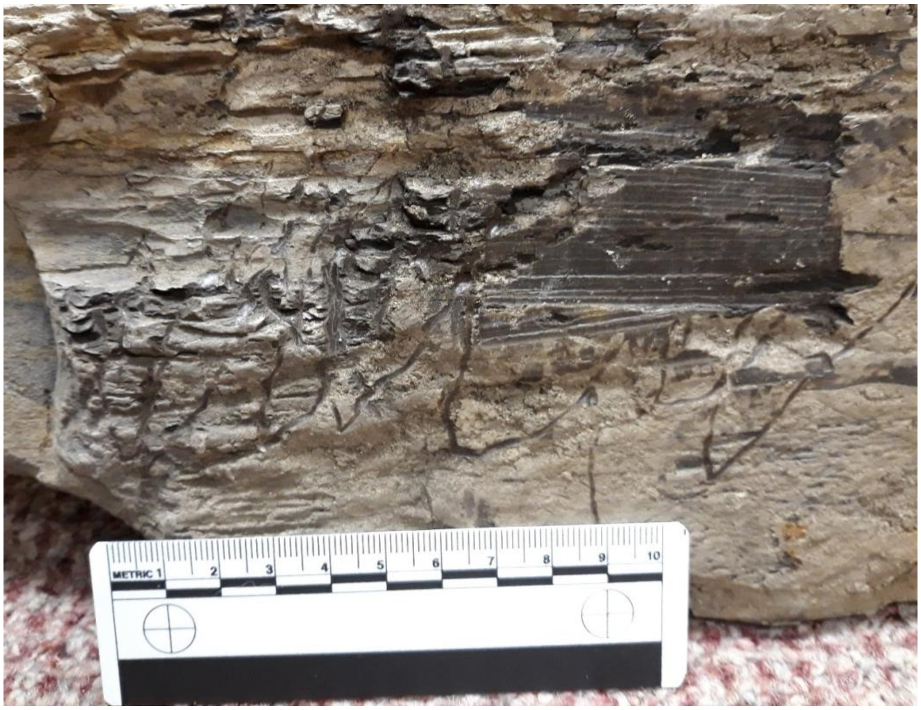

Figure S10. Wood tissue-remained plant fossils from the Chattanooga Shale of the Southern Appalachian Basin.

REFERENCES

1 Gelpi, E., Schneider, H., Mann, J. & Oro, J. Hydrocarbons of geochemical significance in microscopic algae. *Phytochemistry* **9**, 603–612 (1970).

2 Shiea, J., Brassell, S. C. & Ward, D. M. Mid-chain branched mono-and dimethyl alkanes in hot spring cyanobacterial mats: a direct biogenic source for branched alkanes in ancient sediments? *Organic Geochemistry* **15**, 223–231 (1990).

3 Lu, H., Peng, P. a. & Sun, Y. Molecular and stable carbon isotopic composition of monomethylalkanes from one oil sand sample: source implications. *Organic Geochemistry* **34**, 745–754 (2003).

4 Flannery, E. N. & George, S. C. Assessing the syngeneity and indigeneity of hydrocarbons in the ~1.4 Ga Velkerri Formation, McArthur Basin, using slice experiments. *Organic Geochemistry* **77**, 115–125 (2014).

5 Granoff, J. A., Gensel, P. G. & Andrews, H. N. A new species of *Pertica* from the Devonian of eastern Canada. *Palaeontographica Abteilung B*, 119–128 (1976).

6 Andrews, H. N., Gensel, P. G. & Kasper, A. E. A new fossil plant of probable intermediate affinities (Trimerophyte–Progymnosperm). *Canadian Journal of Botany* **53**, 1719–1728 (1975).

7 Kasper Jr, A. E. & Andrews Jr, H. N. *Pertica*, a new genus of Devonian plants from northern Maine. *American Journal of Botany*, 897–911 (1972).

8 Kräusel, R. & Weyland, H. Beiträge zur Kenntnis der devonflora. II. *Abh Senckenb Natforsch Ges* **40**, 115–155 (1926).

9 Kräusel, R. & Weyland, H. Beiträge zur Kenntnis der devonflora. III. *Abh Senckenb Natforsch Ges* **41**, 315–360 (1929).

10 Leclercq, S. *Calamophyton primaevum*: the complex morphology of its fertile appendage. *American Journal of Botany*, 773–781 (1969).

11 Schweitzer, H.-J. Die Mitteldevon-Flora von Lindlar (Rheinland). 4. Filicinae---*Calamophyton primaevum* Kräusel & Weyland. *Palaeontographica Abteilung B*, 117–150 (1973).

12 Leclercq, S. & Andrews Jr, H. N. *Calamophyton bicephalum*, a new species from the Middle Devonian of Belgium. *Annals of the Missouri Botanical Garden*, 1–23 (1960).

13 Bonamo, P. M. & Banks, H. P. *Calamophyton* in the Middle Devonian of New York State. *American Journal of Botany*, 778–791 (1966).

14 Stein, W. E. & Hueber, F. M. The anatomy of *Pseudosporochnus*: *P. hueberi* from the Devonian of New York. *Review of palaeobotany and palynology* **60**, 311–359 (1989).

15 Matten, L. C. The Givetian flora from Cairo, New York: *Rhacophyton*, *Triloboxylon* and *Cladoxylon*. *Botanical Journal of the Linnean Society* **68**, 303–318 (1974).

16 Scheckler, S. E. *Rhymokalon*, a new plant with cladoxylalean anatomy from the Upper Devonian of New York State. *Canadian Journal of Botany* **53**, 25–38 (1975).

17 Stockmans, F. *Végétaux mésodévoniens récoltés aux confins du massif du Brabant: Belgique*. **159** 3–49 (1968).
18 Stein, W. E., Mannolini, F., Hernick, L. V., Landing, E. & Berry, C. M. Giant cladoxylopsid trees resolve the enigma of the Earth's earliest forest stumps at Gilboa. *Nature* **446**, 904–908 (2007).
19 Dannenhoffer, J. M. & Bonamo, P. M. *Rellimia thomsonii* from the Givetian of New York: secondary growth in three orders of branching. *American Journal of Botany* **76**, 1312–1325 (1989).
20 Stein, W. E., Berry, C. M., Hernick, L. V. & Mannolini, F. Surprisingly complex community discovered in the mid-Devonian fossil forest at Gilboa. *Nature* **483**, 78–81, doi:10.1038/nature10819 (2012).
21 Arnold, C. A. Observations on fossil plants from the Devonian of eastern North America, V. *Hyenia banksii*, sp. nov. . *Contributions from Museum of Paleontology University of Michigan* **6**, 53–57 (1941).
22 Fairon-Demaret, M. & Berry, C. M. A reconsideration of *Hyenia elegans* Kräusel et Weyland and Hyeniacomplexa'Leclercq: two Middle Devonian cladoxylopsids from western Europe. *International journal of plant sciences* **161**, 473–494 (2000).
23 Fairon-Demaret, M. & Li, C.-S. *Lorophyton goense* gen. et sp. nov. from the Lower Givetian of Belgium and a discussion of the Middle Devonian Cladoxylopsida. *Review of Palaeobotany and Palynology* **77**, 1–22 (1993).
24 Obrhel, J. Die Flora der Srbsko-Schichten (Givet) des mittelböhmischen Devons. *Sbornik Ustredniho ustavu geologickeho* **26**, 7–46 (1961).
25 Leclercq, S. & Banks, H. P. *Pseudosporochnus nodosus* sp. nov., a Middle Devonian plant with cladoxylalean affinities. *Palaeontographica Abteilung B* **110**, 1–34 (1962).
26 Berry, C. M. & Fairon-Demaret, M. A reinvestigation of the cladoxylopsid *Pseudosporochnus nodosus* Leclercq et Banks from the Middle Devonian of Goé, Belgium. *International Journal of Plant Sciences* **158**, 350–372 (1997).
27 Neumann-Mahlkau, P. Neue Pflanzenfunde und ihre Bedeutung für die Grenze Unterdevon/Mitteldevon am Nordwestabfall des Hohen Venn. *Fortschr. Geol. Rheinld. u. Westf* **9**, 877–882 (1965).
28 Arnold, C. A. Structure and relationships of some Middle Devonian plants from western New York. *American Journal of Botany* **27**, 57–63 (1940).
29 Arnold, C. A. Observations on fossil plants from the Devonian of eastern North America. VI. *Xenocladia medullosina* Arnold. *Contributions from Museum of Paleontology University of Michigan* **9**, 297–309 (1952).
30 Lessuise, A. & Fairon-Demaret, M. Le gisement à plantes de Niaster (Aywaille, Belgique): repère biostratigraphique nouveau aux abords de la limite Couvinien-Givetien. *Annales de la Société géologique de Belgique* **103**, 157–181 (1980).
31 Scott, D. New discoveries in the Middle Devonian Flora of Germany. *New Phytologist*

25, 373–379 (1926).
32 Kräusel, V. R. & Weyland, H. Pflanzenreste aus dem Devon III. Uber Hyenia Nath. *Senckenbergiana* **14**, 274–280 (1932).
33 Schweitzer, H.-J. & Matten, L. C. *Aneurophyton germanicum* and *Protopteridium thomsonii* from the Middle Devonian of Germany. *Palaeontographica Abteilung B* **184**, 64–106 (1983).
34 Stein, W. E. SUNY-Binghamton Paleobiology Collection. (From Paleobiology Database/Fossilworks; https://paleobiodb.org/) (July 2017). (2002).
35 http://www.devoniantimes.org/ (Accessed in 07/2017).
36 Curry, R. P. Miospores from the Upper Devonian (Frasnian) Greenland Gap Group, Allegheny Front, Maryland, West Virginia and Virginia, USA. *Review of Palaeobotany and Palynology* **20**, 119–131 (1975).
37 Beck, C. B. *Studies in the Genus Callixylon*. Thesis, Cornell Univ., New York (1952).
38 Beck, C. B. A new root species of *Callixylon*. *American Journal of Botany* **40**, 226–233 (1953).
39 Serlin, B. S. & Banks, H. P. Morphology and anatomy of *Aneurophyton*, a progymnosperm from the Late Devonian of New York. *Palaeontographica Americana* **8**, 343–359 (1978).
40 Dawson, J. W. The fossil plants of the Erian (Devonian) and Upper Silurian Formations of Canada. *Geological Survey of Canada, Montreal*, 95–140 (1882).
41 Carluccio, L. M., Hueber, F. M. & Banks, H. P. *Archaeopteris macilenta*, anatomy and morphology of its frond. *American Journal of Botany* **53**, 719-730 (1966).
42 Traverse, A. & Schuyler, A. Palynostratigraphy of the Catskill and part of the Chemung Magnafacies, southern New York State, USA. *Courier Forschungsinstitut Senckenberg* **169**, 261–274 (1994).
43 Arnold, C. A. Observations on fossil plants from the Devonian of eastern North America. IV. Plant remains from the Catskill Delta deposits of northern Pennsylvania and southern New York. *Contributions from the Museum of Paleontology, University of Michigan* **5**, 271–314 (1939).
44 Chaloner, W. & Pettitt, J. A seed megaspore from the Devonian of Canada. *Palaeontology* **7**, 29–36 (1964).
45 Beck, C. B. The identity of *Archaeopteris* and *Callixylon*. *Brittonia* **12**, 351-368 (1960).
46 Gensel, P. G. & Barnett-Lawrence, M. Plant megafossils from the Escuminac Formation. In *Devonian Fishes and Plants of Miguasha, Quebec, Canada: Verlag Dr. Friedrich Pfeil, München* (eds Schultze, H. P. and Gloutier, R.) 79–90 (1996).
47 Scheckler, S. E. Ontogeny of progymnosperms. II. Shoots of upper Devonian Archaeopteridales. *Canadian Journal of Botany* **56**, 3136–3170 (1978).
48 Arnold, C. A. The so-called branch impressions of *Callixylon newberryi* (Dn) Elkins and Wieland and the conditions of their preservation. *The Journal of Geology* **42**, 71–76 (1934).
49 Baker, R. C. The age and fossils of the Olentangy Shale of central Ohio. *American*

Journal of Science **240**, 137–143 (1942).

50 Andrews, H. N., Phillips, T. L. & Radforth, N. W. Paleobotanical studies in Arctic Canada: I. *Archaeopteris* from Ellesmere Island. *Canadian Journal of Botany* **43**, 545–556 (1965).

51 Arnold, C. A. Bark structure of *Callixylon*. *Botanical Gazette* **90**, 427–431 (1930).

52 Skog, J. E. *Callixylon* wood from the Greenland Gap Group (Upper Devonian) of southwestern Virginia. *Virginia journal of science* **34**, 58–64 (1983).

53 Stockmans, F. Végétaux du Dévonien supérieur de la Belgique. *Musée royal d'histoire naturelle de Belgique* **110**, 3–85 (1948).

54 Scheckler, S. E. Geology, floristics and paleoecology of Late Devonian coal swamps from Appalachian Laurentia (USA). *Annales de la Société géologique de Belgique* **109**, 209–222 (1986).

55 Fairon-Demaret, M. *Dorinnotheca streelii* Fairon-Demaret, gen. et sp. nov., a new early seed plant from the upper Famennian of Belgium. *Review of Palaeobotany and Palynology* **93**, 217–233 (1996).

56 Meyer-Berthaud, B., Soria, A. & Decombeix, A.-L. The land plant cover in the Devonian: a reassessment of the evolution of the tree habit. *Geological Society, London, Special Publications* **339**, 59–70 (2010).

57 Cressler, W. L. Plant paleoecology of the Late Devonian Red Hill locality, north-central Pennsylvania, an *Archaeopteris*-dominated wetland plant community and early tetrapod site. *Geological Society of America Special Papers* **399**, 79–102 (2006).

58 CresslerI, W. L. & Pfefferkorn, H. W. A Late Devonian isoetalean lycopsid, *Otzinachsonia beerboweri*, gen. et sp. nov., from north-central Pennsylvania, USA. *American journal of botany* **92**, 1131–1140 (2005).

59 Doweld, A. *Jurinodendron*—a new replacement name for *Cyclostigma* S. Haughton ex O. Heer, 1871 (Lycopodiophyta). *Paleontological Journal* **35**, 218–221 (2001).

60 Read, C. B. & Campbell, G. Preliminary account of the New Albany Shale flora. *American Midland Naturalist* **21**, 435–453 (1939).

61 Read, C. B. Pennsylvanian floral zones and floral provinces. *The Journal of Geology* **55**, 271–279 (1947).

62 Cross, A. T. & Hoskins, J. H. Paleobotany of the Devonian-Mississippian black shales. *Journal of Paleontology*, 713–728 (1951).

63 Read, C. B. The flora of the New Albany shale: Part 1, *Diichnia kentuckiensis*, a new representative of the Calamopityeae. *USGS Professional Paper* **185-H**, 149–161 (1936).

64 Phillips, T. L., Andrews, H. N. & Gensel, P. G. Two heterosporous species of *Archaeopteris* from the Upper Devonian of West Virginia. *Palaeontographica Abteilung B* **139**, 47–71 (1972).

65 Fairon-Demaret, M. & Leponce, I. Leaf dimorphism in *Archaeopteris roemeriana* (Progymnosperm): further early fossil evidence of shoot dorsiventrality. *American*

Journal of Botany **88**, 729–735 (2001).
66 Cornet, B., Phillips, T. L. & Andrews, H. N. The morphology and variation in *Rhacophyton ceratangium* from the Upper Devonian and its bearing on frond evolution. *Palaeontographica Abteilung B* **158**, 105–129 (1976).
67 Chitaley, S. On the occurrence of *Prototaxites* in the Cleveland Black Shale of Ohio, USA. *Review of Palaeobotany and Palynology* **72**, 257–271 (1992).
68 Arnold, C. A. Some new forms and new occurrences of fossil plants from the Middle and Upper Devonian of New York State. *Bulletin of Buffalo Society of Natural Sciences* **17**, 1–12 (1935).
69 Stubblefield, S. P., Taylor, T. N. & Beck, C. B. Studies of paleozoic fungi. IV. Wood-decaying fungi in *Callixylon newberryi* from the upper Devonian. *American Journal of Botany*, **72**, 1765–1774 (1985).
70 Campbell, J. *Callixylon* from the Upper Devonian of northwestern Alberta. *American Journal of Botany* **50**, 648–652 (1963).
71 Conant, L. C. & Swanson, V. E. Chattanooga Shale and related rocks of central Tennessee and nearby areas. *USGS Professional Paper* **357** (1961).
72 Prestianni, C., Hilton, J. & Cressler, W. Were All Devonian Seeds Cupulate? A Reinvestigation of *Pseudosporogonites hallei*, *Xenotheca bertrandii*, and *Aglosperma* spp. *International Journal of Plant Sciences* **174**, 832–851 (2013).
73 Mintz, J. S., Driese, S. G. & White, J. D. Environmental and Ecological Variability of Middle Devonian (Givetian) Forests in Appalachian Basin Paleosols, New York, United States. *Palaios* **25**, 85-96, doi:10.2110/palo.2009.p09-086r (2010).
74 Berry, C. M. & Mashal, J. E. A. Lycopsid forests in the early Late Devonian paleoequatorial zone of Svalbard. *Geology* **43**, 1043–1046 (2015).
75 Cornet, L., Gerrienne, P., Meyer-Berthaud, B., & Prestianni, C. A Middle Devonian *Callixylon* (Archaeopteridales) from Ronquières, Belgium. *Review of Palaeobotany and Palynology*, **183**, 1–8, doi:10.1016/j.revpalbo.2012.07.004 (2012).
